# All-Polymer Solar Cells and Photodetectors with Improved Stability Enabled by Terpolymers Containing Antioxidant Side Chains

**DOI:** 10.1007/s40820-023-01114-5

**Published:** 2023-05-29

**Authors:** Chunyang Zhang, Ao Song, Qiri Huang, Yunhao Cao, Zuiyi Zhong, Youcai Liang, Kai Zhang, Chunchen Liu, Fei Huang, Yong Cao

**Affiliations:** Institute of Polymer Optoelectronic Materials and Devices, State Key Laboratory of Luminescent Materials and Devices, South China University of Technology, Guangzhou, 510640 People’s Republic of China

**Keywords:** Organic photovoltaics, Device operational stability, All-polymer solar cell, Organic photodetector, Antioxidant

## Abstract

It is of vital importance to improve the long-term and photostability of organic photovoltaics, including organic solar cells (OSCs) and organic photodetectors (OPDs), for their ultimate industrialization. Herein, two series of terpolymers featuring with an antioxidant butylated hydroxytoluene (BHT)-terminated side chain, PTzBI-EHp-BTBHTx and N2200-BTBHTx (*x* = 0.05, 0.1, 0.2), are designed and synthesized. It was found that incorporating appropriate ratio of benzothiadiazole (BT) with BHT side chains on the conjugated backbone would induce negligible effect on the molecular weight, absorption spectra and energy levels of polymers, however, which would obviously enhance the photostability of these polymers. Consequently, all-polymer solar cells (all-PSCs) and photodetectors were fabricated, and the all-PSC based on PTzBI-EHp-BTBHT0.05: N2200 realized an optimal power conversion efficiency (PCE) approaching ~ 10%, outperforming the device based on pristine PTzBI-EHp: N2200. Impressively, the all-PSCs based on BHT-featuring terpolymers displayed alleviated PCEs degradation under continuous irradiation for 300 h due to the improved morphological and photostability of active layers. The OPDs based on BHT-featuring terpolymers achieved a lower dark current at − 0.1 bias, which could be stabilized even after irradiation over 400 h. This study provides a feasible approach to develop terpolymers with antioxidant efficacy for improving the lifetime of OSCs and OPDs.

**Supplementary Information:**

The online version contains supplementary material available at 10.1007/s40820-023-01114-5.

## Highlights

- Two series of terpolymers with improved photostability were realized by the introduction of appropriate ratio of antioxidant butylated hydroxytoluene unit containing side chains.
- All-polymer solar cells and organic photodetectors (OPDs) with these terpolymers as both donors and acceptors have been prepared with simultaneously improved efficiency and stability.
- A feasible approach to develop terpolymers with antioxidant efficacy for improving the lifetime of organic solar cells and OPDs is proposed.

**Supplementary Information:**

The online version contains supplementary material available at 10.1007/s40820-023-01114-5.

## Graphical abstract

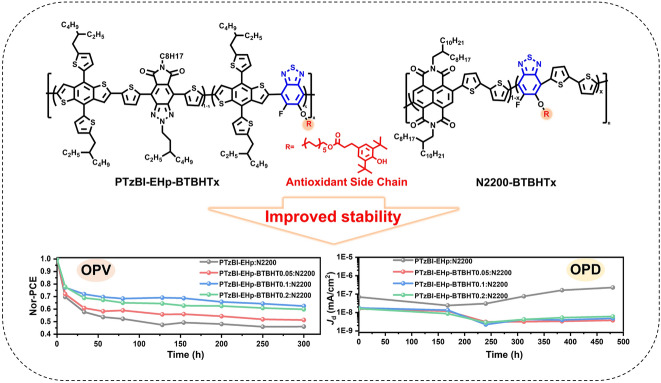

## Introduction

Organic photovoltaics based on organic semiconductors, including organic solar cells (OSCs) and organic photodetectors (OPDs), have attracted extensive attention owing to their merits of light-weight, flexibility, semi-transparency and large-scale processability. With printed electronics techniques such as spray-coating, stamping, screen-printing, inkjet printing, roll-to-roll processing, it is now possible to produce large-area OPDs at a very competitive cost and with promising performance compared to inorganic counterparts [1–5]. Meanwhile, the power conversion efficiency (PCE) of OSCs has soared up to over 19% [6], demonstrating great potential for commercial application in near future. Despite the excited achievements on improving output performance of OSCs and OPDs, devices stability is another one of the most important bottlenecks that need to be overcome for their ultimate industrialization [7, 8]. Therefore, it is of great significance to explore novel materials to prolong the operating lifetime of OSCs and OPDs [9].

Generally, it has been recognized that the OSCs and OPDs are vulnerable to oxygen and moisture when they are exposed to ambient atmosphere, resulting in the degradation of device performance over time. Due to the inferior stability of organic semiconductors compared to inorganic ones, the devices degradation will also be accelerated during operation under light irradiation. It was found that the performance degradation was mainly originated from deterioration of conjugated materials caused by the radical reactions [7, 10–12]. Therefore, antioxidant additives, such as dibutylhydroxytoluene (BHT), have been added to the active layer as radical scavengers to inhibit the degradation of conjugated materials, resulting in improved stability of OSCs [13–15]. Besides the devices instability caused by H_2_O, O_2_ and solar irradiation, the morphology instability under storage or operating has been recognized as another main reason for the degradation of devices [16]. For non-fullerene small molecule acceptors (NFSMAs)-based OSCs, the self-aggregation of NFSMAs will damage the optimal morphology and suppress the charge separation and transport efficiency, leading to the gradual decrease in device performance [17]. The planar heterojunction (PHJ) strategy with layer-by-layer (LBL) fabricated active layer was recently proposed to improve morphology stability [18], however, which will increase the manufacturing procedures of devices. Notably, although adding antioxidant additives can effectively improve the photostability of OSCs, the additives will inevitably induce the morphology instability [19, 20]. Additionally, the exploration of effective strategies to improve stability of OPDs is still rarely reported. Therefore, it is necessary to develop novel conjugated materials for improving stability of OSCs as well as OPDs [21, 22].

All-polymer solar cells (all-PSCs) or photodetectors with polymers as both donor and acceptor have been proved an effective approach to improve the morphological stability [23, 24]. Herein, the antioxidant BHT-featuring side chain is attached on the benzothiadiazole (BT) unit, affording a conjugated block BTBHT with antioxidant efficacy. Then, appropriate molar ratios of the BTBHT moiety are incorporated to the backbone of the widely used polymer donor PTzBI-EHp and polymer acceptor N2200, affording two series of novel terpolymers, PTzBI-EHp-BTBHTx and N2200-BTBHTx (*x* = 0, 0.05, 0.1, 0.2), respectively. It was found that the introduction of BTBHT moiety on conjugated backbone would enhance the photostability without sacrificing the morphological stability of blend films. Consequently, the all-PSC based on PTzBI-EHp-BTBHT0.1: N2200 achieved an optimal PCE of 9.96%, and the devices based on polymers with BTBHT moiety exhibited higher PCEs retention after continuous light irradiation for 300 h under ambient atmosphere. Investigations revealed that the introduction of BTBHT moiety could significantly suppress the trap-assisted bimolecular recombination under irradiation, guaranteeing the efficient charge separation and transport within active layers and thus improving the device photostability. In addition, all-polymer OPDs have been fabricated, and lower dark current density (*J*_d_) at − 0.1 V bias and higher specific detectivity (D*) were achieved for OPDs based on PTzBI-EHp-BTBHTx and N2200-BTBHTx (*x* = 0.05, 0.1, 0.2). Noteworthy, OPDs based on PTzBI-EHp-BTBHTx: N2200 (*x* = 0.05, 0.1, 0.2) showed almost negligible *J*_d_ increase under continuous irradiation over 400 h, significantly outperforming the device based on PTzBI-EHp: N2200. As a result, photoplethysmography (PPG) sensors for real-time heart rate detection were carried out with our OPDs based on PTzBI-EHp-BTBHTx: N2200, and considerable detection resolution and sensitivity could be obtained. This study provides a feasible molecular design strategy to develop conjugated polymers for all-PSCs and OPDs with simultaneously improved morphological and photostability.

## Experimental Section

### Materials Synthesis

The design of our new polymer donors and acceptors is derived from the PTzBI-EHp and N2200, respectively. Varied molar percentages of BT units with antioxidant BHT group-terminated side chain were used as the third component monomer for terpolymerization to afford the target terpolymers, PTzBI-EHp-BTBHTx and N2200-BTBHTx (*x* = 0, 0.05, 0.1, 0.2) (Scheme S1). All terpolymers are synthesized straightforwardly, and the intermediates and resulting polymers were characterized with nuclear magnetic resonance (NMR) spectra (Figs. S1–S19). The copolymerized ratios of BT units in both polymers have been quantitatively confirmed by analyzing the ^1^H NMR spectra evolution of polymers with gradually increased ratio of BT units (Figs. S12 and S17). More materials synthesis details were described in the Supporting Information. The resulting terpolymers are readily soluble in common organic solvents such as chloroform (CF), chlorobenzene (CB) and 1, 2-dichlorobenzene (*o*-DCB) at room temperature, enabling their solution processability for all-PSCs and OPDs.

### Materials Characterization

Molecular weights of polymers were measured on an Agilent Technologies PL-GPC 220 high-temperature chromatograph in 1,2,4-trichlorobenzene at 150 °C using a calibration curve of polystyrene standards. The ^1^H/^13^C NMR test of compounds 2, 3 and M1 was conducted on a Bruker AV-400/500 MHz spectrometer in CDCl_3_ at 25 °C. The ^1^H NMR test of PTzBI-BTBHTx was conducted on a Bruker AV-400 MHz spectrometer in CDCl_3_ at 25 °C, while the test of N2200-BTBHTx was conducted on a Bruker AV-400 MHz spectrometer in 1,1,2,2-C_2_D_2_Cl_4_ at 80 °C due to its relatively inferior solubility in CDCl_3_. Differential scanning calorimetry (DSC) curves were acquired from DSC 2500 with filmed samples of 2–3 mg under nitrogen flow at heating and cooling rates of 10 °C min^−1^. Thermogravimetric analysis (TGA) measurements were measured on TG209F1 with powdered samples of 2–3 mg under nitrogen flow at heating and cooling rates of 10 °C min^−1^. UV–Vis–NIR absorption spectra were recorded on a SHIMADZU UV-3600 spectrophotometer from 300 to 1000 nm. Cyclic voltammetry (CV) was measured on a CHI 660A Electrochemical Workstation with Ferrocene/ferrocenium (Fc/Fc^+^) as the calibration standard, and the potential of saturated calomel electrodes (SCE) was internally calibrated as 0.39 V. The energy level was calculated according to the following equations: *E*_HOMO_ = − (*E*_ox_ + 4.41) eV and *E*_LUMO_ = − (*E*_re_ + 4.41) eV, where *E*_ox_ and *E*_re_ are the onset oxidation potential and onset reduction potential relative to Hg/Hg_2_Cl_2_, respectively.

### Device Fabrication

The OSC devices were fabricated in a conventional device configuration of ITO/PEDOT:PSS/Active layer/PFNBr/Ag. The OPD devices were based on an inverted device structure of ITO/ZnO/Active layer/MoO_3_/Ag. The patterned ITO glass substrates were cleaned sequentially by sonication with detergent once, deionized water three times, and then isopropanol twice, and then dried at 65 °C in a baking oven overnight. After UV-ozone treatment for 4 min, for the fabrication of OSC, the ITO substrates were coated with PEDOT:PSS (CLEVIOSTM P VP AI 4083 from Heraeus) at 3500 rpm for 30 s. For OPD device, the ITO substrates were coated with ZnO at 3000 rpm for 30 s. The ZnO solution was synthesized by mixing 0.4 g of zinc acetate dehydrate in 110 μL of ehtanolamine and 4 mL of methoxyethanol. After annealed in the air at 150 °C on a hot plate for 15 min, the substrates were transferred into a nitrogen-filled protected glove box. The solution of polymer donor: acceptor (2:1, w/w, dissolved by 2-methyltetrahydrofuran (Me-THF), with a total concentration of 9 mg mL^−1^) were spin-coated at 3000 rpm to afford an ~ 110 nm active layer for OSC devices and 1000 rpm to obtain ~ 230 nm active layer for OPD devices, respectively. Sequentially, the active layers were thermally annealed for 10 min. Then, a thin layer of PFNBr was coated from its methanol solution (1 mg mL^−1^) onto the active layer to form an electron transport layer for OSCs, and a 10 nm of MoO_3_ was deposited on the active layer to form a hole transport layer for OPDs. Lastly, 100 nm Ag was thermally deposited on top of all devices through a mask under a vacuum of ~ 1 × 10^–7^ mbar. The effective area of the devices was 0.0516 cm^2^. More characterization methods for the devices are described in the Supporting Information.

## Results and Discussion

### Molecular Weights, Photophysical and Electrochemical Properties of Terpolymers

**Fig. 1:**
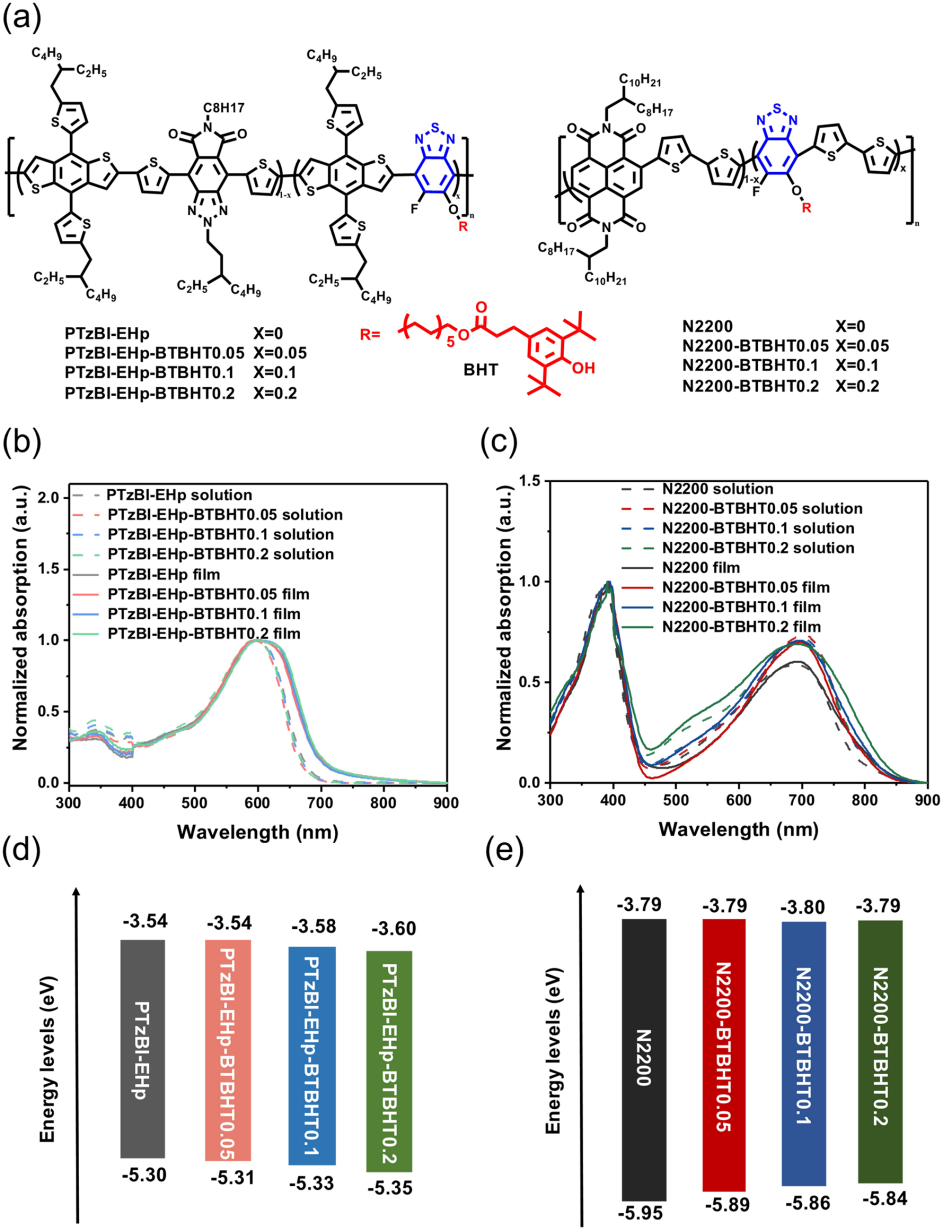
**a** Chemical structures, **b, c** absorption spectra in solutions and as films and **d, e** energy-level alignment of polymer donors PTzBI-EHp-BTBHTx and polymer acceptors N2200-BTBHTx

The molecular structures of the polymer donors PTzBI-EHp-BTBHTx and acceptors N2200-BTBHTx are shown in Fig. 1a. Varied ratios of BT units with BHT-featuring side chain were incorporated to enhance the stability of polymers due to the antioxidant ability of the BHT group. It was found that polymers PTzBI-EHp-BTBHTx exhibited number-average molecular weights (*M*_n_) in the range of 31–45 kDa with the polydispersion index (PDI) of ~ 2.1, while polymers N2200-BTBHTx exhibited the *M*_n_ of 98–114 kDa with PDI of ~ 2.3. The corresponding parameters are summarized in Table 1, and their characteristic curves are shown in Figs. S20 and S21. These results demonstrated that the appropriate ratios of BTBHT units would not significantly affect the molecular weights of prepared polymers, which could eliminate the molecular weights influence on polymers properties and device performance.

**Table 1:** Molecular weights, optical properties and frontier molecular orbital energy levels of polymer donors PTzBI-EHp-BTBHT*x* (*x* = 0, 0.05, 0.1, 0.2)

Polymer	*M*_n_ (kDa)	PDI	$\lambda_{{{\text{max}}}}^{{\text{a}}}$ (nm)	$\lambda_{{{\text{onset}}}}^{{\text{a}}}$ (nm)	$\lambda_{{{\text{max}}}}^{{\text{b}}}$ (nm)	$\lambda_{{{\text{onset}}}}^{{\text{b}}}$ (nm)	$E_{{\text{g}}}^{{\text{opt c}}} \left( {{\text{eV}}} \right)$	$E_{{{\text{ox}}}}$ (V)	$E_{{{\text{HOMO}}}}^{{\text{d}}}$ (eV)	$E_{{{\text{LUMO}}}}^{{\text{e}}}$ (eV)
PTzBI-EHp	42.8	2.1	594	678	599	704	1.76	0.89	− 5.30	− 3.54
PTzBI-EHp-BTBHT0.05	31.4	2.1	593	673	596	702	1.77	0.90	− 5.31	− 3.54
PTzBI-EHp-BTBHT0.1	37.1	2.3	597	676	604	707	1.75	0.92	− 5.33	− 3.58
PTzBI-EHp-BTBHT0.2	45.4	2.5	593	676	599	710	1.75	0.94	− 5.35	− 3.60

^a^In solutions

^b^In films

^c^$$E_{{\text{g}}}^{{{\text{opt}} }}$$ = 1240/$$\lambda_{{{\text{onset}}}}$$

^d^$$E_{{{\text{HOMO}}}} = - \left( {E_{{{\text{ox}}}} - E_{{\left( {\frac{{{\text{Fc}}}}{{{\text{Fc}}^{ + } }}} \right)}} + 4.8} \right)$$ eV

^e^$$E_{{{\text{LUMO}}}} = E_{{{\text{HOMO}}}} + E_{{\text{g}}}^{{{\text{opt}}}}$$

The UV–Vis–NIR absorption spectra of PTzBI-EHp-BTBHTx and N2200-BTBHTx in Me-THF solutions and as thin films are shown in Fig. 1b, c. The relevant data are listed in Tables 1 and 2, respectively. PTzBI-EHp-BTBHTx (*x* = 0.05, 0.1, 0.2) display the almost identical solution and film absorption compared to their pristine polymer PTzBI-EHp, and a red-shift of the absorption edge could be observed for the films. By contrast, N2200-BTBHTx (*x* = 0.05, 0.1, 0.2) exhibited solution and film absorption with the similar absorption peaks compared to their pristine polymer N2200, and no red-shifted absorption could be observed for the films. Notably, the intensity enhancement of the charge-transfer absorption band (600–800 nm) could be observed for N2200-BTBHTx (*x* = 0.05, 0.1, 0.2) compared to N2200, and an increase in 450–560 nm could be observed for N2200-BTBHT0.2 due to the superposition effect of the BTBHT moiety absorption. The optical bandgap (${{E}_{\mathrm{g}}}^{\mathrm{opt}}$) of ~ 1.75 and ~ 1.49 eV could be, respectively, calculated for PTzBI-EHp-BTBHTx and N2200-BTBHTx from their absorption onsets.

**Table 2:** Molecular weights, optical properties and frontier molecular orbital energy levels of acceptor polymers N2200-BTBHT*x* (*x* = 0, 0.05, 0.1, 0.2)

Polymer	*M*_n_ (kDa)	PDI	$\lambda_{{{\text{max}}}}^{{\text{a}}}$ (nm)	$\lambda_{{{\text{onset}}}}^{{\text{a}}}$ (nm)	$\lambda_{{{\text{max}}}}^{{\text{b}}}$ (nm)	$\lambda_{{{\text{onset}}}}^{{\text{b}}}$ (nm)	$E_{{\text{g}}}^{{\text{opt c}}} \left( {{\text{eV}}} \right)$	$E_{{{\text{ox}}}}$ (V)	$E_{{{\text{re}}}}$ (V)	$E_{{{\text{HOMO}}}}^{{\text{d}}}$ (eV)	$E_{{{\text{LUMO}}}}^{{\text{e}}}$ (eV)
N2200	98	2.5	390/690	800	391/692	829	1.50	1.54	− 0.62	− 5.95	− 3.79
N2200-BTBHT0.05	106	2.3	390/699	813	391/697	830	1.49	1.48	− 0.62	− 5.89	− 3.79
N2200-BTBHT0.1	114	2.4	391/699	813	395/696	831	1.49	1.45	− 0.61	− 5.86	− 3.80
N2200-BTBHT0.2	111	2.3	391/696	813	390/693	835	1.48	1.43	− 0.62	− 5.84	− 3.79

^a^In solutions

^b^In films

^c^$$E_{{\text{g}}}^{{{\text{opt}} }}$$ = 1240/$$\lambda_{{{\text{onset}}}}$$

^d^$$E_{{{\text{HOMO}}}} = - \left( {E_{{{\text{ox}}}} - E_{{\left( {\frac{{{\text{Fc}}}}{{{\text{Fc}}^{ + } }}} \right)}} + 4.8} \right)$$ eV

^e^$$E_{{{\text{LUMO}}}} = - \left( {E_{{{\text{re}}}} - E_{{\left( {\frac{{{\text{Fc}}}}{{{\text{Fc}}^{ + } }}} \right)}} + 4.8} \right)$$ eV

The electrochemical properties of all polymers were investigated by CV measurements (Fig. S22). As shown in Fig. 1d and Table 1, the highest occupied molecular orbital (HOMO) and the lowest unoccupied molecular orbital (LUMO) of PTzBI-EHp-BTBHTx and N2200-BTBHTx (*x* = 0.05, 0.1, 0.2) were similar to those of pristine PTzBI-EHp and N2200, respectively (Fig. 1e and Table 2). These results suggest that the introduction of reasonable ratio of BTBHT unit will not significantly change the electronic energy levels of obtained terpolymers. The complementary absorption and matched energy levels of PTzBI-EHp-BTBHTx and N2200-BTBHTx make them suitable polymer donor and acceptor, respectively, for OSCs and OPDs.

To gain more insights of the materials properties, the thermal properties of all polymers were evaluated by TGA and DSC measurements (Fig. S23). The 5% weight loss temperatures over 400 °C of both PTzBI-EHp-BTBHTx and N2200-BTBHTx indicate that all polymers have good thermal stability. The DSC results of polymer donors PTzBI-EHp-BTBHTx show no obvious melting or crystallization peaks in the temperature range of 25–300 °C, indicating their amorphous characteristics. Differed from PTzBI-EHp-BTBHTx, all polymers N2200-BTBHTx exhibit melting and crystallization peaks, and the melting temperature is gradually lowered with increasing the BTBHT moiety ratio from 0 to 0.2, indicating the introduction of BTBHT moiety may decrease the crystallinity of N2200.

### Photostability and Morphological Properties of Blend Films

**Fig. 2:**
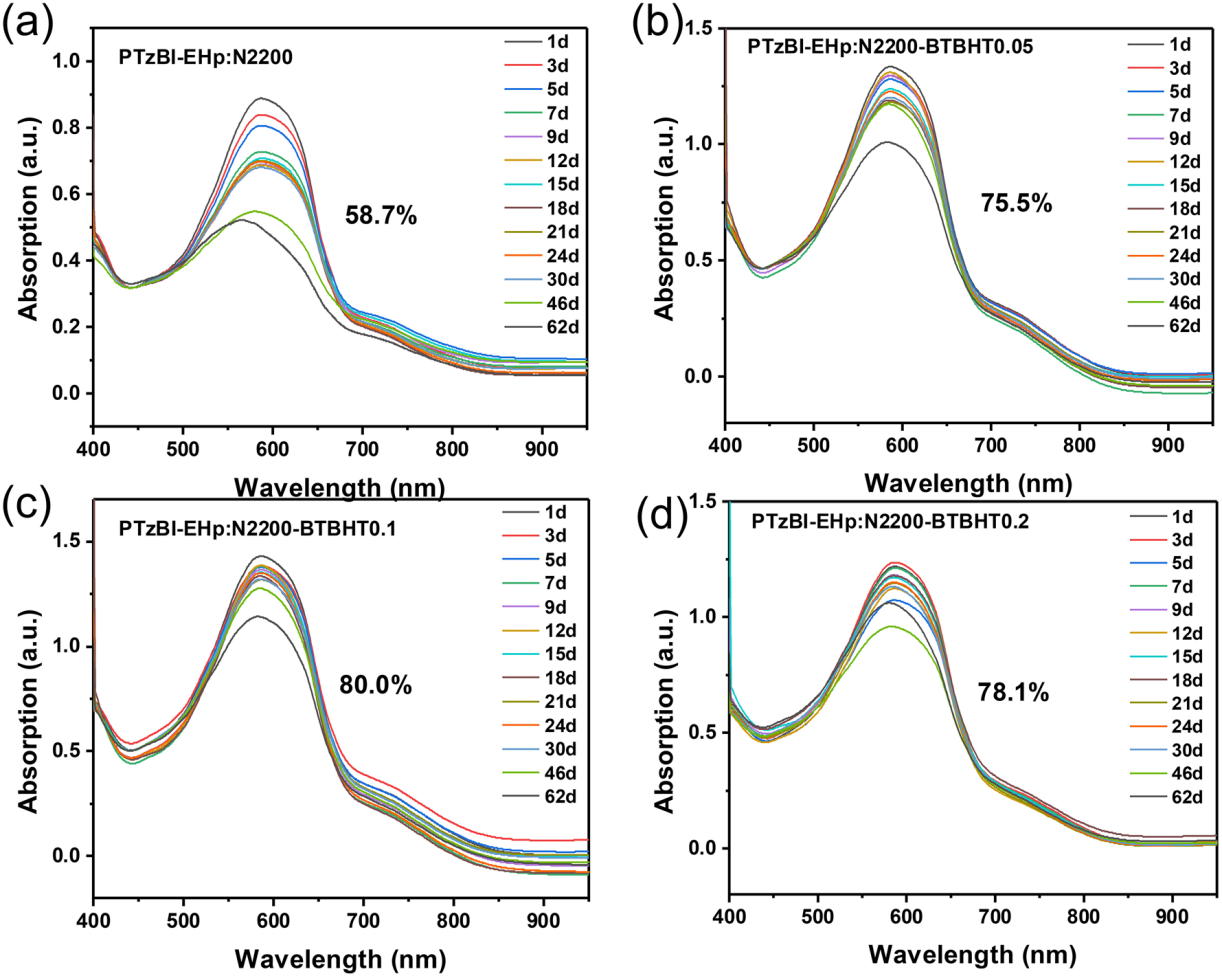
UV–Vis–NIR absorption spectra changes of PTzBI-EHp: N2200-BTBHT*x* (*x* = 0, 0.05, 0.1, 0.2) blend films with continuous light irradiation under ambient condition: **a** PTzBI-EHp: N2200, **b** PTzBI-EHp: N2200-BTBHT0.05, **c** PTzBI-EHp: N2200-BTBHT0.1 and **d** PTzBI-EHp: N2200-BTBHT0.2

A preliminary investigation on the stability of the polymers is to monitor their UV–Vis-NIR absorption spectra change during continuous aging under light and ambient condition. As shown in Fig. 2, for blend films of PTzBI-EHp: N2200-BTBHTx (*x* = 0, 0.05, 0.1, 0.2), the absorption intensity decrease (500–700 nm) after 62 days of continuous light irradiation under ambient condition can be effectively suppressed when reasonable ratios of BTBHT moiety are incorporated. Particularly, 80% of the peak absorption can be remained for the PTzBI-EHp: N2200-BTBHT0.1 blend film, significantly higher than 58.7% for the PTzBI-EHp: N2200 film. Similarly, the absorption intensity decrease can be effectively decelerated for PTzBI-EHp-BTBHTx: N2200 (*x* = 0.05, 0.1, 0.2)-based blend films after light irradiation under ambient condition. (Fig. S24). It should be noted that the absorption band in 500–650 nm of blend films can mainly ascribed to the PTzBI-EHp, while the absorption band in 680–800 nm is mainly ascribed to the N2200 and the weak absorption intensity in 680–800 nm is presumably due to the lower absorption coefficient of N2200 than PTzBI-EHp. As demonstrated in previous work, for PTzBI-EHp: N2200-BTBHTx blend films, the BHT groups could eliminate the radicals in both PTzBI-EHp and N2200 polymer chains, resulting in the suppressed deterioration of PTzBI-EHp even though the BHT group was attached into N2200 [20].

**Fig. 3:**
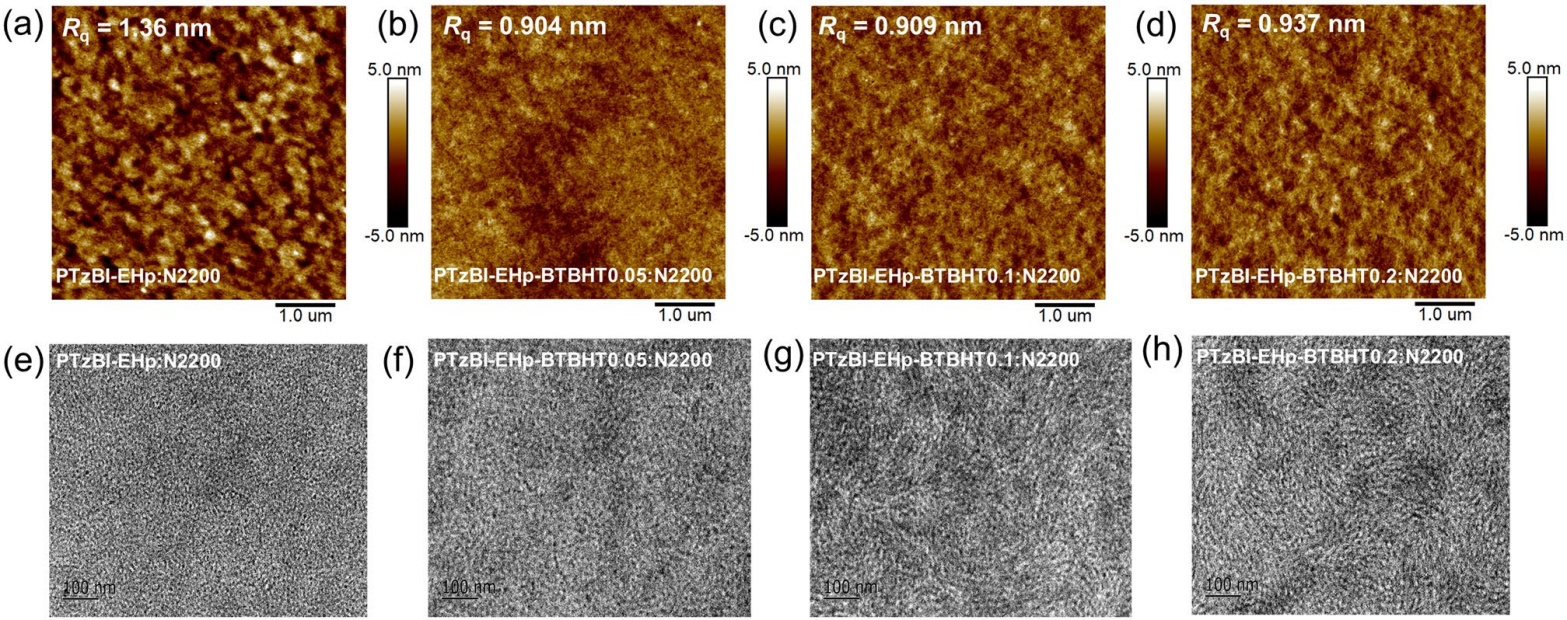
**a–d** AFM (5 μm × 5 μm) and **e–h** TEM images (1 μm × 1 μm) of PTzBI-EHp-BTBHT*x*: N2200 (*x* = 0, 0.05, 0.1, 0.2) blend films

The influence of incorporating antioxidant moiety BTBHT on the morphology of PTzBI-EHp: N2200 blend films was analyzed by atomic force microscopy (AFM) and transmission electron microscopy (TEM) (Figs. 3 and S25-S26). As shown in Figs. 3a–d and S25, the root mean square (RMS) roughness values are slightly decreased to a similar value for the surface of both PTzBI-EHp-BTBHTx: N2200 and PTzBI-EHp: N2200-BTBHTx blend films. It suggests that the introduction of antioxidant moiety by terpolymerization will generate a slight improvement on the surface morphology of blend films, and more ratios of BTBHT units would not significantly affect the surface morphology of blend films. As shown in the TEM images in Fig. S26, the phase separation degree and the bicontinuous interpenetrating network structure of PTzBI-EHp: N2200-BTBHTx (*x* = 0, 0.05, 0.1, 0.2) blend films are similar. In contrast, a fibrous structure can be observed in the PTzBI-EHp-BTBHT0.1: N2200 and PTzBI-EHp-BTBHT0.2: N2200 blend films (Fig. 3e–h), suggesting that an appropriate ratio of BTBHT moiety in the PTzBI-EHp may be beneficial for forming a better blend films morphology and thus improving performance of OSCs and OPDs.

### Photovoltaic Properties

**Fig. 4:**
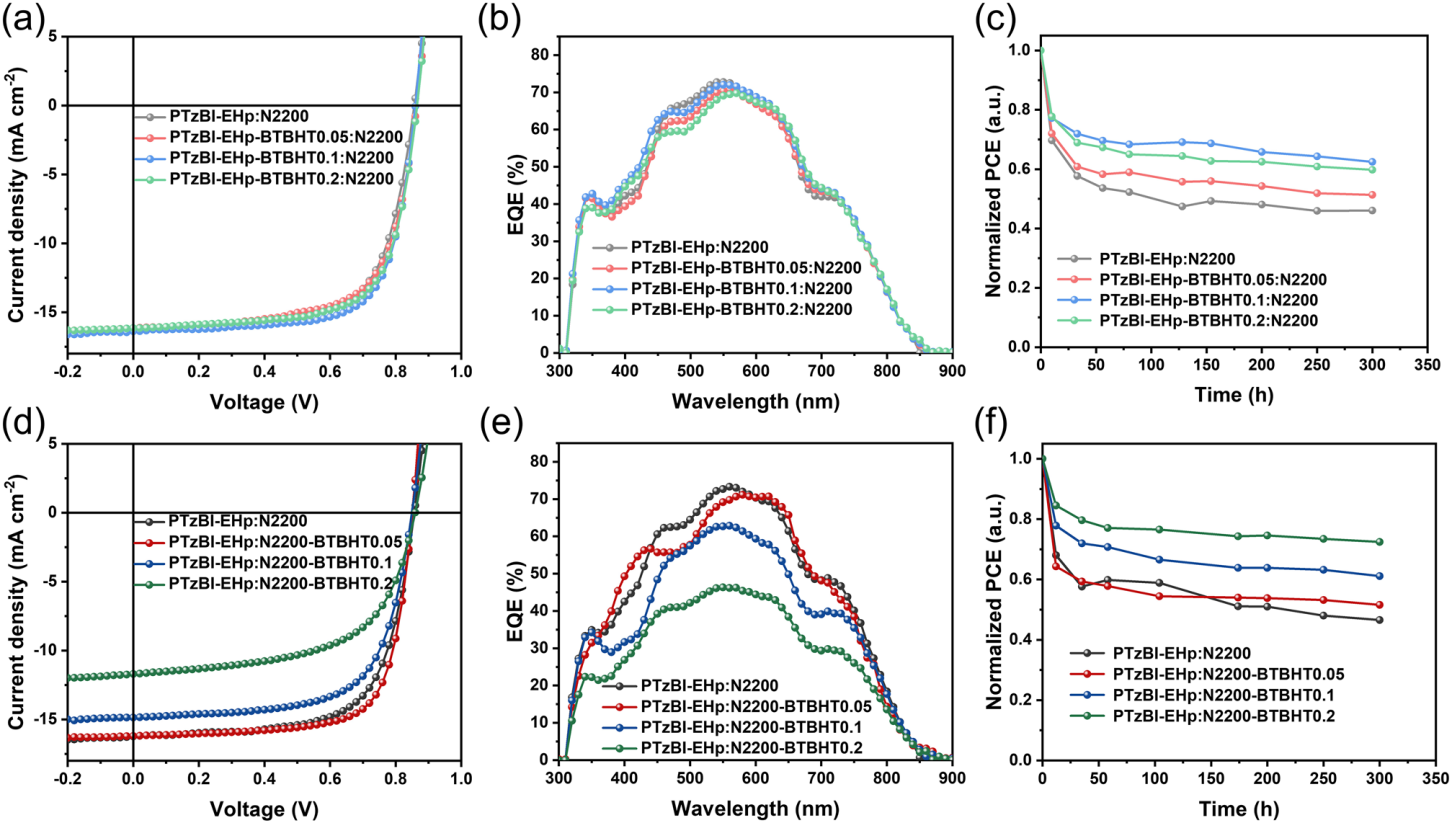
*J–V* characteristics of all-PSCs based on **a** PTzBI-EHp-BTBHT*x* (*x* = 0, 0.05, 0.1, 0.2): N2200 and **d** PTzBI-EHp: N2200-BTBHT*x* (*x *= 0, 0.05, 0.1, 0.2), respectively. EQE curves of all-PSCs based on **b** PTzBI-EHp-BTBHT*x* (*x* = 0, 0.05, 0.1, 0.2): N2200 and **e** PTzBI-EHp: N2200-BTBHT*x* (*x* = 0, 0.05, 0.1, 0.2), respectively. The PCE track under 300 h of irradiation at inert atmosphere for all-PSCs based on **c** PTzBI-EHp-BTBHT*x* (*x* = 0, 0.05, 0.1, 0.2): N2200 and **f** PTzBI-EHp: N2200-BTBHT*x* (*x* = 0, 0.05, 0.1, 0.2)

The photovoltaic performance of all-PSCs with synthesized polymers as active layers was evaluated. The statistical summary of photovoltaic parameters is shown in Table 3. All the devices were tested under the irradiation of AM1.5 G, 100 mW cm^−2^. As displayed in Fig. 4a, all-PSCs based on PTzBI-EHp-BTBHTx: N2200 exhibited similar open-circuit voltages (*V*_oc_s) and short-circuit current densities (*J*_sc_s), which are in good accordance with the *J*_sc_s calculated by external quantum efficiency (EQE) spectral integral (Fig. 4b). The highest fill factor (FF) of 70.87% is achieved for the device based on PTzBI-EHp-BTBHT0.1: N2200, which can be ascribed to the superior morphology as shown in Fig. 3g. As a result, the highest optimal PCE of 9.96% can be realized for PTzBI-EHp-BTBHT0.1: N2200-based all-PSC. For all-PSCs based on PTzBI-EHp: N2200-BTBHTx, similar *V*_oc_s can be obtained for all devices; however, significantly decreased *J*_sc_s and FF values can be observed for PTzBI-EHp: N2200-BTBHTx (*x* = 0.1, 0.2)-based all-PSCs (Fig. 4d, e). Consequently, the device based on PTzBI-EHp: N2200-BTBHT0.05 achieves the highest optimal PCE of 9.97%, much higher than 8.37% and 5.92% of devices based on PTzBI-EHp: N2200-BTBHT0.1 and PTzBI-EHp: N2200-BTBHT0.2, respectively. This result indicates that the all-PSCs performance is more sensitive to the copolymerization ratio of BTBHT moiety into the N2200, which is presumably due to the more significant effect of incorporating BTBHT moiety on the crystallinity of N2200. To be noted here, OSC devices based on PTzBI-EHp-BTBHTx (*x* = 0.1, 0.2): N2200-BTBHTx (*x* = 0.05, 0.1) system were further fabricated, which showed no enhanced photovoltaic performance (as shown in Table S1). Therefore, we focus our discussion mainly on PTzBI-EHp-BTBHTx: N2200-BTBHTx system in this work.

**Table 3:** Photovoltaic parameters of all-PSCs

Active layer	*V*_oc_ (V)	*J*_sc_ (mA cm^−2^)	FF (%)	PCE_MAX_ (%)
PTzBI-EHp:N2200	0.86 ± 0.00 0.86	16.04 ± 0.19 16.24	66.89 ± 0.42 67.32	9.20 ± 0.13 9.34
PTzBI-EHp-BTBHT0.05:N2200	0.86 ± 0.00 0.87	15.81 ± 0.12 15.95	66.12 ± 0.26 66.36	9.02 ± 0.03 9.04
PTzBI-EHp-BTBHT0.1:N2200	0.86 ± 0.00 0.86	16.38 ± 0.06 16.44	70.66 ± 0.17 70.87	9.93 ± 0.03 9.96
PTzBI-EHp-BTBHT0.2:N2200	0.86 ± 0.00 0.87	16.08 ± 0.15 16.18	69.19 ± 0.60 69.88	9.60 ± 0.02 9.62
PTzBI-EHp:N2200-BTBHT0.05	0.85 ± 0.00 0.85	15.97 ± 0.19 16.20	72.33 ± 0.92 73.35	9.83 ± 0.13 9.97
PTzBI-EHp:N2200-BTBHT0.1	0.85 ± 0.00 0.85	14.28 ± 0.52 14.86	65.64 ± 1.30 67.15	7.96 ± 0.45 8.37
PTzBI-EHp:N2200-BTBHT0.2	0.86 ± 0.00 0.86	11.44 ± 0.34 11.70	59.26 ± 0.79 60.13	5.81 ± 0.12 5.92

To verify the speculations that the polymers featured with antioxidant BHT groups can improve the stability of the OSCs in the air condition, encapsulated OSCs were firstly aged in the air at room temperature under continuous light irradiation with a solar simulator (an intensity of 100 mW cm^−2^ LED). The PCE values of devices were tracked during the aging experiment period until 300 h as shown in Fig. 4c, f. All data were normalized to their initial PCE value at 0 h. After 300-h aging, the PCE retention rates of all-PSCs based on PTzBI-EHp: N2200, PTzBI-EHp-BTBHT0.05: N2200, PTzBI-EHp-BTBHT0.1: N2200 and PTzBI-EHp-BTBHT0.2: N2200 were recorded as 46.0%, 51.3%, 62.4% and 59.8%, respectively. The PCE retention rates of 46.6%, 51.6%, 61.1% and 72.4% could be observed for all-PSCs based on PTzBI-EHp: N2200, PTzBI-EHp: N2200-BTBHT0.05, PTzBI-EHp: N2200-BTBHT0.1 and PTzBI-EHp: N2200-BTBHT0.2, respectively. The higher PCE retention rate of 62.4% for PTzBI-EHP-BTBHT0.1: N2200-based device and 72.4% for PTzBI-EHp: N2200-BTBHT0.2-based device than ~ 46% for PTzBI-EHp: N2200-based device demonstrates that incorporating antioxidant BTBHT moiety is effective to improve the all-PSCs stability under light and air condition. In addition, the long-term stability of unencapsulated OSCs under light irradiation and H_2_O and O_2_ ambient conditions was explored. As shown in Fig. S27, when exposed to light and ambient conditions, all unencapsulated devices exhibit faster degradation rates than those encapsulated devices. This further indicates that the entry of H_2_O and O_2_ in the atmosphere is the main cause of photooxidative degradation of OSCs. Notably, comparing with the device based on PTzBI-EHp: N2200, the devices based on both PTzBI-EHp-BTBHTx and N2200-BTBHTx (*x* = 0.05, 0.1, 0.2) exhibited a slower PCE degradation after 6 h of exposure to light irradiation and ambient condition, further validating the efficacy of copolymerizing BTBHT moiety for preventing the free radical degradation pathway of conjugated polymers and eventually improving devices long-term stability. Moreover, the devices with antioxidant BHT groups also demonstrated superior long-term thermal stability. Device stability with different content of BHT groups was evaluated without encapsulation at 80 °C in N_2_-filled glovebox. As shown in Fig. S28, the trends of the PCE values are consistent with that of long-term stability of OSCs under light irradiation, indicating that the introduction of BHT groups also plays a positive role in the morphological stability.

### Charge-Carrier Dissociation and Recombination Dynamics

**Fig. 5:**
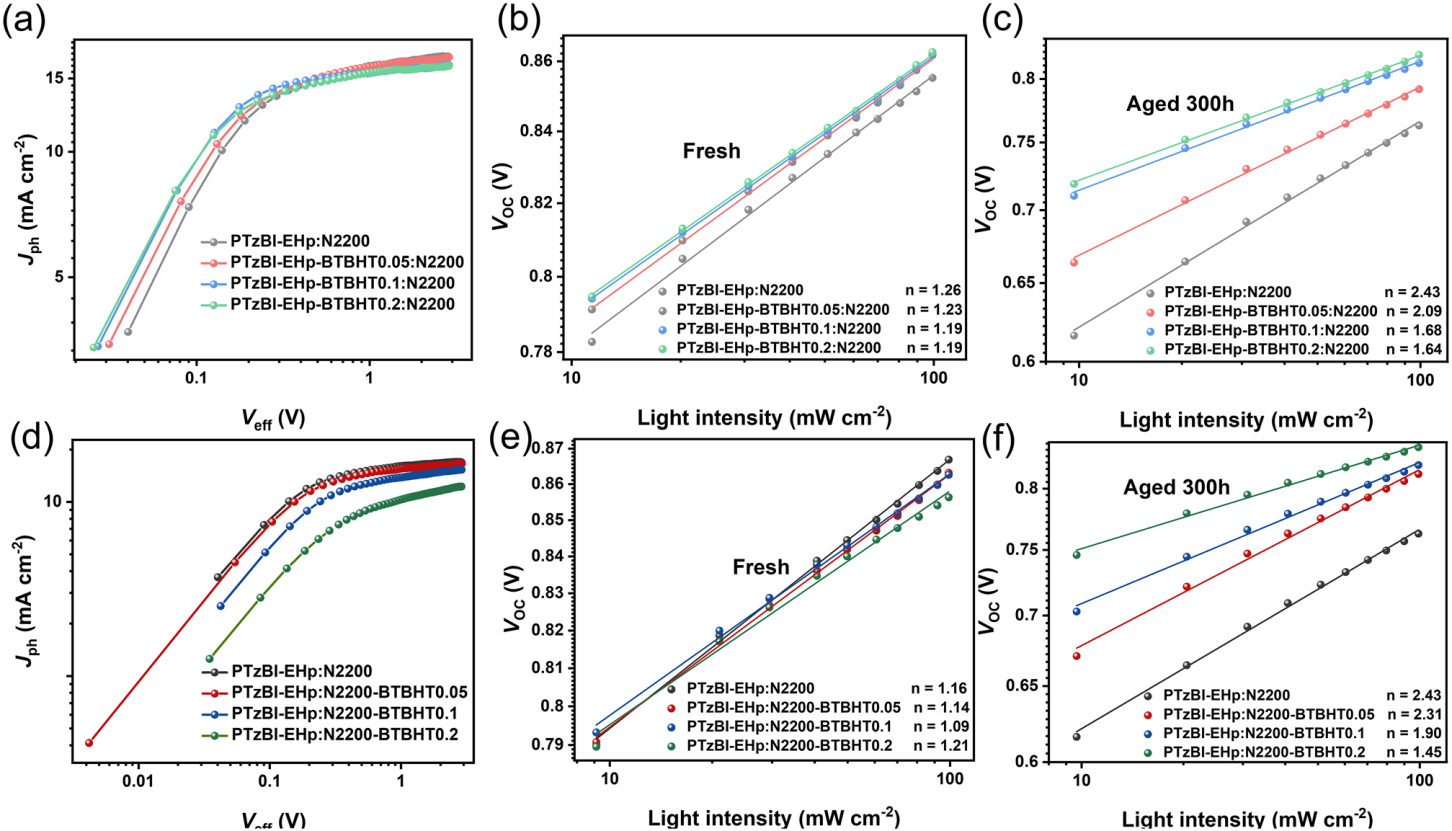
**a, d** The charge dissociation efficiency $P(\mathrm{E},\mathrm{ T})$ of the OSCs. **b, e** Measurement of *V*_oc_ versus light intensity of the fresh OSCs. **c, f** Measurement of *V*_oc_ versus light intensity of the OSCs after aging for 300 h

The exciton dissociation probability $P(\mathrm{E},\mathrm{ T})$ was estimated by plotting photocurrent (${J}_{\mathrm{ph}}$) versus effective voltage (${V}_{\mathrm{eff}}$), and the maximum exciton generation rates (*G*_max_) are defined as ${J}_{\mathrm{sat}}$*/qL*, where *q* represents elementary charge, *L* is the film thickness, and ${J}_{\mathrm{sat}}$ denotes the saturation current [25]. The photocurrent can reach saturation within a large effective voltage region, and most excitons can be separated to produce charge carriers under the action of an applied electric field, which are then collected by the electrode. As shown in Fig. 5a, for all-PSCs based on PTzBI-EHp-BTBHTx (*x* = 0, 0.05, 0.1, 0.2): N2200, all devices show similar *G*_max_ values of about 1.0 × 10^28^ m^−3^ s^−1^ and $P(\mathrm{E},\mathrm{ T})$ values of more than 93% under the condition of short circuit, indicating that all devices have effective exciton generation and dissociation. Particularly, the device based on PTzBI-EHp-BTBHT0.1: N2200 displays the highest $P(\mathrm{E},\mathrm{ T})$ value of 96.28%, indicating that the introduction of BTBHT moiety into PTzBI-EHp has little effect on charge generation and collection in PTzBI-EHp-BTBHTx (*x* = 0, 0.05, 0.1, 0.2): N2200 based all-PSCs. These results are consistent with the aforementioned similar FF values, which is recognized to be closely correlated to the exciton generation and charge recombination process [26]. Meanwhile, for all-PSCs based on PTzBI-EHp: N2200-BTBHTx (*x* = 0, 0.05, 0.1, 0.2), the *G*_max_ value and $P(\mathrm{E},\mathrm{ T})$ value shows an obvious trend of decline under the short-circuit condition for devices based on polymers N2200 with increased BTBHT ratio (Fig. 5d). The significantly lower *G*_max_ values and $P(\mathrm{E},\mathrm{ T})$ values of PTzBI-EHp: N2200-BTBHTx (*x* = 0.1, 0.2)-based devices indicate the inferior exciton generation and dissociation within active layers, which can well explain the lower FF and *J*_sc_ values in these two devices. The relevant $P(\mathrm{E},\mathrm{ T})$ and *G*_max_ values are summarized in Table S2.

Non-geminate recombination dominates in OSCs and can be divided into two molecular recombination and trap-assisted recombination [27]. In order to study the carrier recombination behavior of these devices, the light intensity (${P}_{\mathrm{light}}$) dependence of *J*_sc_ was first determined. According to the power law ${J}_{\mathrm{sc}}\propto {P}_{\mathrm{light}}^{a}$, where *a* is an exponential factor and the exponent *a* represents the degree of bimolecular recombination. If no bimolecular recombination exists in the device, the exponent $\mathrm{\alpha }$ should be close to 1 [28]. The values of the fitting lines for OSCs based on PTzBI-EHp, PTzBI-EHp-BTBHT0.05, PTzBI-EHp-BTBHT0.1 and PTzBI-EHp-BTBHT0.2 were 0.980, 0.991, 0.989 and 0.990, respectively (Fig. S29). The devices offered an similar *a* value even after 300 h of aging. These results show that the introduction of BTBHT has little effect on the bimolecular recombination loss in these devices. As expected, we get similar results in OSCs based on PTzBI-EHp: N2200-BTBHTx systems. Furthermore, the relationship of *V*_oc_ as a function of ${P}_{\mathrm{light}}$ was analyzed. The relationship between *V*_oc_ and ${P}_{\mathrm{light}}$ can be expressed as:${V}_{\mathrm{oc}}\propto \frac{nkT}{q}\mathrm{ln}({P}_{\mathrm{light}})$, where $k$ is Boltzmann constant, $T$ is absolute temperature and $q$ is elementary charge. When the slope close to 2 $kT$/$q$, trap-assisted recombination is involved [29]. As shown in Fig. 5b, the slopes of PTzBI-EHp-BTBHT0.05-, PTzBI-EHp-BTBHT0.1- and PTzBI-EHp-BTBHT0.2-based devices are 1.23, 1.19 and 1.19 $kT$/$q$, respectively, slightly lower than the slopes of PTzBI-EHp-based device (1.26 $kT$/$q$). It is worth noting that after a period of aging, the slopes of the PTzBI-EHp-BTBHT0.05-, PTzBI-EHp-BTBHT0.1- and PTzBI-EHp-BTBHT0.2-based devices are significantly smaller than that of the PTzBI-EHp-based device (Fig. 5c), suggesting that the trap-assisted recombination is effectively inhibited in devices based on polymers with BTBHT moiety. The similar results could be found for OSCs based on PTzBI-EHp: N2200-BTBHTx systems (Fig. 5e, f), indicating that the device containing BHT group plays a significant role in inhibiting the trap-assisted recombination during photooxidative degradation, which may be the reason for the slower PCEs attenuation of OSCs in the photooxidative stability test.

### OPD Performance

**Fig. 6:**
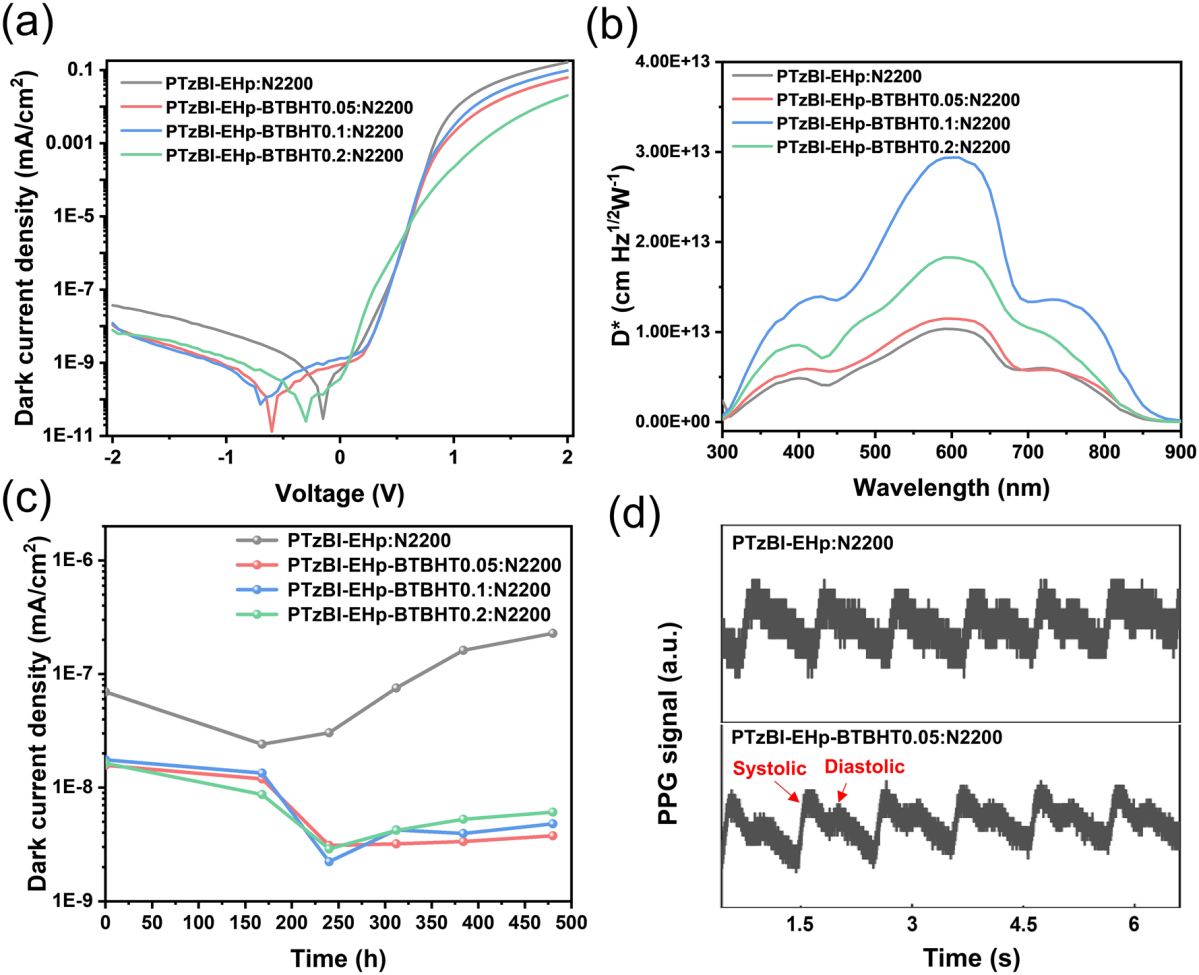
**a** *J–V* curves of the OPDs under dark condition; **b** specific detectivity obtain from *J*_d_ of OPDs in this work; **c** the stability of *J*_d_ of OPDs after aging for several days.** d** PPG signal fluctuations of OPD devices after aging

To further check the photovoltaic performance of the synthesized polymers, organic photodetectors for light detecting were also fabricated. Figures 6a and S30a presented the relevant duck current density (*J*_d_)-voltage (*V*) curves of OPDs based on the pristine and antioxidant BHT-modified polymers with the same processing conditions and device structure. It is obvious that a suppressed dark current density by almost one order of magnitude could be achieved for OPDs based on PTzBI-EHp-BTBHTx (*x* = 0.05, 0.1, 0.2) and N2200-BTBHTx (*x* = 0.05, 0.1, 0.2) compared to that based on PTzBI-EHp and N2200. Notably, the low *J*_d_ of the OPD devices is beneficial for achieving high-quality signals for broad utility in practical applications [25–28]. Furthermore, dark current can be extracted according to the following Shockley diode equation [30]:

1 $$J_{{\text{d}}} = J_{0} \left[ {\exp \left( {\frac{qV}{{nkT}}} \right) - 1} \right] + \frac{V}{{R_{{{\text{sh}}}} }}$$

where $J_{0}$ is the saturated dark current density, $n$ is ideality factor, $k$ is Boltzmann’s constant, $T$ is temperature, and $R_{{{\text{sh}}}}$ is the shut resistance. As shown in Table S3, the increased $R_{{{\text{sh}}}}$ of OPDs based on the BTBHT-featuring polymers is consistent with the reduced *J*_d_ under reverse biases, indicating an improved ability to block the reverse injection current [30–32].

Another key figure-of-merit of OPD devises is the specific detectivity (D*) [33, 34, 35], which depicts a photodetector’s sensitivity to weak light signals and given by:

2 $$D^{*} = \frac{{R_{{{\text{res}}}} \sqrt A }}{{S_{{{\text{noise}}}} }}$$

where $R_{{{\text{res}}}}$ is spectral responsivity and the corresponding data is shown in Fig. S31, A is the area of device ($A$ = 0.0516 cm^2^ in this work), $S_{{{\text{noise}}}}$ is the noise current. Notably, a thick-film strategy was applied for the fabrication of OPD devices for the suppression of *J*_d_. Therefore, the EQE of the OPDs are relatively low compared to the OSC devices as a result of the low charge mobility of organic semiconductors and severe charge recombination in thick photoactive layers. The value of D* can be simply expressed by $R_{{{\text{res}}}}$/(2q*J*_d_)^1/2^ as the shot noise is dominant under reverse bias [36, 37]. More details are described in Note S1. As shown in Figs. 6b and S32, the D* values under − 0.1 V bias of devices based on BTBHT-featuring polymers are significantly higher than the control device, achieving the values over 10^13^ Jones (Jones = cm Hz^1/2^ W^−1^) at 350–750 nm. The maximum D* of 2.94 × 10^13^ and 5.35 × 10^13^ Jones at 600 nm could be obtained for OPDs based on PTzBI-EHp-BTBHT0.1: N2200 and PTzBI-EHp: N2200-BTBHT0.1, respectively.

For the OPD based on BTBHT-featuring polymers PTzBI-EHp-BTBHTx and N2200-BTBHTx, we further evaluate the stability by monitoring the value of the dark current density during the aging procedure. The unencapsulated OPD devices were aged in the air condition at room temperature. Figures 6c and S30b display the variation curves of *J*_d_ under reverse bias of − 2 V for all the OPD devices. For devices based on PTzBI-EHp: N2200, the *J*_d_ value starts to remarkably increase after aging over 250 h. However, the *J*_d_ values of devices based on PTzBI-EHp-BTBHTx (*x* = 0.05, 0.1, 0.2) show no obvious increase and can be stabilized at order lower than 10^–8^ mA cm^−2^, demonstrating the effectiveness of BTBHT units for preventing the degradation of OPDs. As for the stability of the dark current density based on polymer acceptors N2200-BTBHTx, to our surprise, the introduction of BTBHT moiety on N2200 has negligible influence on long-term *J*_d_ stability (Fig. S30b). We attribute this decreasing in OPD performance to the high noise signals of OPD devices after aging based on polymer acceptors N2200-BTBHTx (Fig. S33).

In addition, PPG sensors for real-time heart rate detection were carried out using our OPDs based on PTzBI-EHp-BTBHTx: N2200 because of its outstanding stability at air condition. PPG of a single red light source (light intensity of 0.72 mW) was recorded by detecting changes in the reflected light from the skin, which infers the arterial pulse wave. As displayed in Figs. 6d and S34, the PPG signals were detected by the OPD devices which were aged for 500 h in the air condition. Notably, the OPDs based on PTzBI-EHp-BTBHT*x*: N2200 (*x* = 0.05, 0.1, 0.2) provided higher detectivity than the control device, facilitating increased resolution of PPG signal with clear systolic and diastolic peak.

## Conclusions

In summary, a general strategy for enhancing the photostability of conjugated polymers was proposed with introducing the antioxidant BHT group on the side chains of terpolymers. Two series of polymer donors and acceptors, PTzBI-EHp-BTBHT*x* (*x* = 0, 0.05, 0.1, 0.2) and N2200-BTBHT*x* (*x* = 0, 0.05, 0.1, 0.2) were successfully synthesized. Appropriate ratio of BTBHT moiety in the conjugated backbone would not significantly affect the molecular weight, absorption and energy levels of polymers. Impressively, comparing with pristine polymer PTzBI-EHp and N2200, BHT-containing polymers exhibit enhanced photostability under continuous light irradiation over 60 days at ambient atmosphere. All-polymer solar cells and photodetectors based on obtained polymers were fabricated. The all-PSC based on PTzBI-EHp-BTBHT0.05: N2200 achieved an optimal PCE reaching ~ 10%, relatively higher than that of PTzBI-EHp: N2200 based cells. Noteworthy, all-PSCs based on polymers with BHT-featuring side chains achieved superior device stability under light irradiation of 300 h. The deep insight demonstrated that the suppressed trap-assisted charge recombination might be the main reason for the enhanced photostability of all-PSCs based on BTH-featuring polymers. In addition, all-polymer photodetectors have been fabricated with these polymers, and OPDs based on BHT-featuring polymers achieved lower *J*_d_ and higher D* than devices based on PTzBI-EHp: N2200. The D* over 10^13^ Jones could be obtained in the region of 350–750 nm for PTzBI-EHp-BTBHT0.05: N2200 based device. Moreover, OPDs based on PTzBI-EHp-BTBHT*x*: N2200 (*x* = 0.05, 0.1, 0.2) realized steady and low *J*_d_ under continuous irradiation over 300 h, whereas the *J*_d_ of PTzBI-EHp: N2200 based device was significantly increased after irradiation over 200 h. This study provides an effective molecular design approach to improve the stability of OSCs and OPDs, which may shed some light on the future material development for the ultimate industrialization of organic photovoltaics.

### Supplementary Information

Below is the link to the electronic supplementary material.

Supplementary file1 (PDF 3778 kb)
