# Supplementary material for: All-Polymer Solar Cells and Photodetectors with Improved Stability Enabled by Terpolymers Containing Antioxidant Side Chains
Source: Nanomicro Lett. 2023 May 29;15:140. doi: 10.1007/s40820-023-01114-5 (PMC10226943; doi:10.1007/s40820-023-01114-5)
Supplement: Supplementary file 1 — Supplementary file1 (PDF 3778 kb) [file 40820_2023_1114_MOESM1_ESM.pdf]

Supporting Information for

All-Polymer Solar Cells and Photodetectors with Improved Stability Enabled by Terpolymers Containing Antioxidant Side Chains

Chunyang Zhang[1, #], Ao Song[1, #], Qiri Huang[1, #], Yunhao Cao[1], Zuiyi Zhong[1], Youcai Liang[1], Kai Zhang[1], Chunchen Liu[1, *], Fei Huang[1, *], Yong Cao[1]

[1]Institute of Polymer Optoelectronic Materials and Devices, State Key Laboratory of Luminescent Materials and Devices, South China University of Technology, Guangzhou 510640, P. R. China

[#] Chunyang Zhang, Ao Song, and Qiri Huang contributed equally to this work.

* Corresponding authors. E-mail: mscliu@scut.edu.cn (Chunchen Liu); msfhuang@scut.edu.cn (Fei Huang)

S1 Materials and Methods

S1.1 Materials

The reagents and solvents were purchased from commercial sources and used as received unless otherwise noted.

S1.2 Synthetic Procedures of Intermediates and Polymers

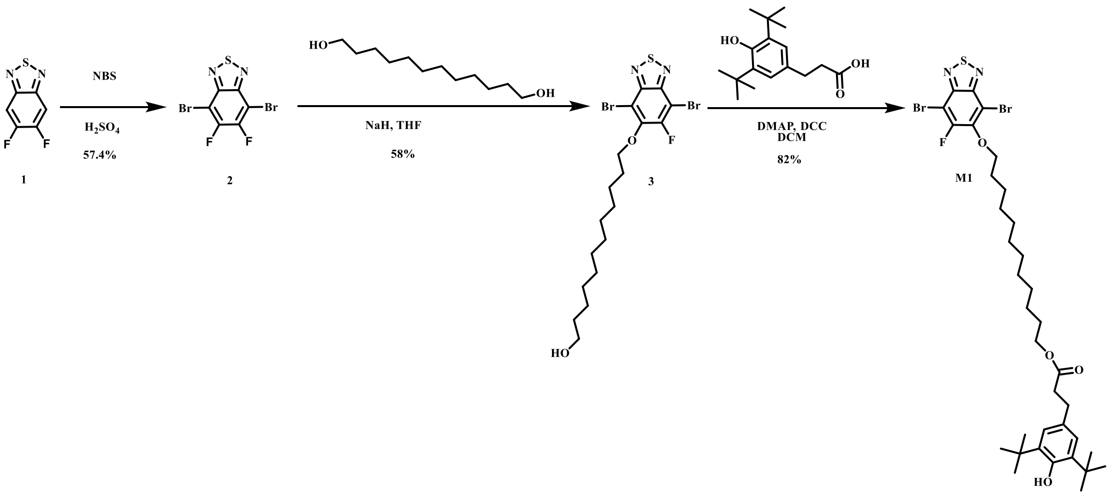

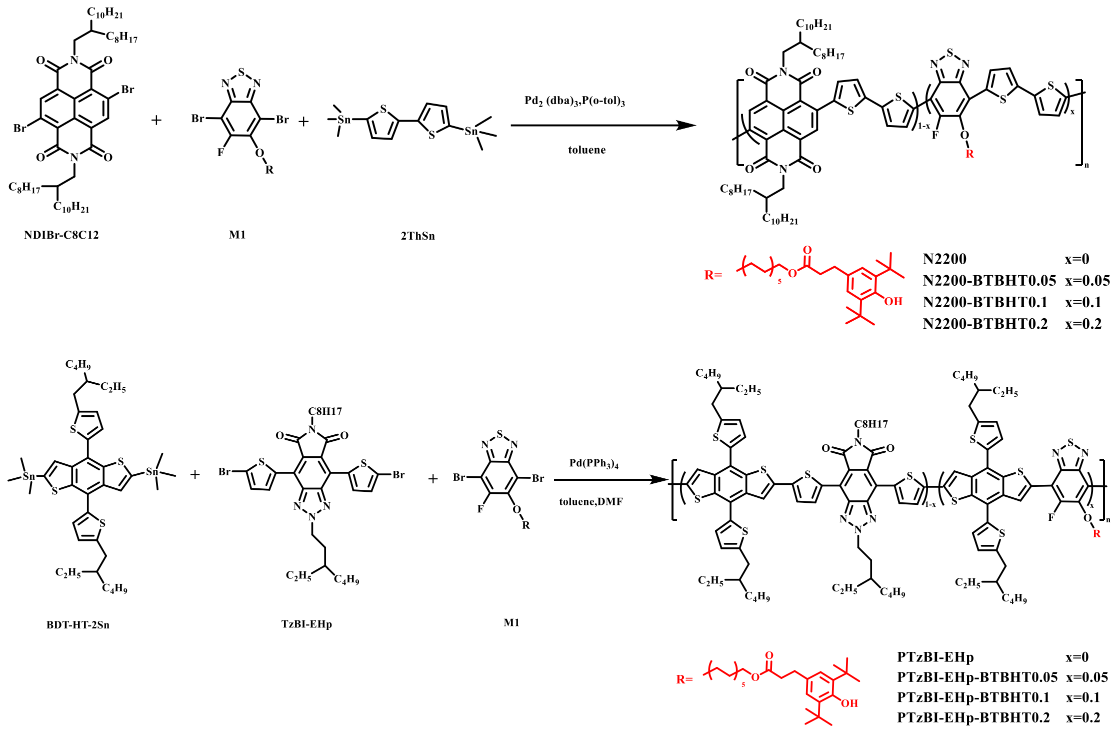

Scheme S1 Synthetic routes of M1, N2200-BTBHT*x* and PTzBI-EHp-BTBHTx

S1.2.1 4,7-Dibromo-5,6-difluorobenzo[c][1,2,5]thiadiazole (2)

Under an argon atmosphere, compound 1 (1g, 5.8 mmol) was dissolved in 50 mL of concentrated sulfuric acid. Then 4.57 g (25.5 mmol) of N-bromosuccinimide (NBS) was added and the reaction mixture was slowly heated to 50 °C for 4 h. The reaction procedure was monitored by thin film chromatography (TLC) in real time. After the complete consumption of compound 1, the mixture was cooled to room temperature and then poured into the ice water. The precipitate was collected by filtration and washed with deionized water. Subsequently, the resulting white solid was dissolved in dichloromethane and washed with saturated brine for 3 times. The organic phase was dried with anhydrous magnesium sulfate, and then concentrated. The product 2 was purified by silica gel column chromatography with a yield of 57%.

^{1}H NMR (400 MHz, Chloroform-d) δ 7.26 (s, 1H).

^{13}C NMR (126 MHz, Chloroform-d) δ 152.94, 152.78, 150.86, 150.69, 148.88, 148.86, 148.84, 99.57, 99.47, 99.40, 99.34, 99.28.

S1.2.2 12-((4,7-dibromo-6-fluorobenzo[c][1,2,5]thiadiazol-5-yl)oxy)dodecan-1-ol (3)

Under an argon atmosphere, 1,12-dodecanediol (0.92 g, 4.5 mmol) was dissolved in 60 mL of tetrahydrofuran solution, and then 60% sodium hydride (0.14 g, 3.6 mmol) was added. The reaction mixture was stirred at 50 °C for 3 h, followed by which compound 2 (1.0 g, 3.0 mmol) was added. The mixture was stirred at 50 °C overnight. The reaction mixture was poured into deionized water, and extracted with dichloromethane. The organic phase was collected, dried with anhydrous magnesium sulfate, and concentrated. The mixture was further purified by column chromatography with

petroleum ether and methylene chloride (1:3 vol%) as eluent. The product was obtained with a yield of 58%.

^{1}H NMR (400 MHz, Chloroform-d) δ 4.24 (t, J = 6.4 Hz, 2H), 3.65 (t, J = 6.7 Hz, 2H), 1.89 (t, J = 7.4 Hz, 2H), 1.42 – 1.23 (m, 19H).

^{13}C NMR (126 MHz, Chloroform-d) δ 157.70, 155.64, 149.98, 149.45, 149.29, 149.14, 149.10, 106.21, 98.66, 98.47, 75.82, 75.78, 75.15, 63.11, 32.81, 30.27, 30.12, 29.59, 29.55, 29.52, 29.43, 29.28, 25.76, 25.75.

S1.2.3 12-((4,7-dibromo-6-fluorobenzo[c][1,2,5]thiadiazol-5-yl)oxy)dodecyl3-(3,5-di-tert-butyl-4-hydroxyphenyl)propanoate(M1)

Under an argon atmosphere, compound 2 (0.9 g, 1.76 mmol) was dissolved in 60 mL of dichloromethane solution. 3-(3,5-di-tert-butyl-4-hydroxyphenyl) propionic acid (0.54 g, 1.93 mmol) and 4-dimethylaminopyridine (0.023 g, 0.193 mmol) were added sequentially. The color of the resulting solution gradually became pale yellow. Then the solution temperature was cooled to 0 °C and *N*, *N'*-dicyclohexyl carbon diimide (0.4 g, 1.93 mmol) was added. The color changed from light yellow to milky white, and the solution was restored to room temperature and stirred overnight. The reaction solution was filtered and the filtrate is collected. The mixture was further purified by column chromatography with petroleum ether and methylene chloride (2:1 vol%) as the eluent. The product M1 was obtained as the pale yellow liquid product with a yield of 82%.

^{1}H NMR (400 MHz, Chloroform-d) δ 6.99 (s, 2H), 5.07 (s, 1H), 4.23 (t, J = 6.5 Hz, 2H), 4.07 (t, J = 6.8 Hz, 2H), 2.87 (t, J = 8.0 Hz, 2H), 2.59 (dd, J = 9.1, 7.0 Hz, 2H), 1.89 (t, J = 7.5 Hz, 2H), 1.62 (t, J = 6.7 Hz, 2H).

^{13}C NMR (126 MHz, Chloroform-d) δ 173.37, 152.13, 149.98, 135.87, 131.16, 124.77, 75.81, 75.77, 64.62, 36.52, 34.30, 31.03, 30.31, 30.12, 29.55, 29.53, 29.51, 29.29, 29.27, 28.65, 25.92, 25.76.

S1.2.4 Synthesis of the PTzBI-EHp-BTBHTx and N2200-BTBHTx (x=0.05, 0.1, 0.2)

For the synthesis of polymer donors PTzBI-EHp-BTBHTx, Pd(PPh3)$_4$ (5.0 mg) was added to a degassed solution of TzBI-EHp (0.1–0.1x mmol), M1 (0.1x mmol), and BDT-HT-2Sn (0.1 mmol) (x = 0 for PTzBI-EHp; x = 0.05 for PTzBI-EHp-BTBHT0.05; x = 0.1 for PTzBI-EHp-BTBHT0.1; x = 0.2 for PTzBI-EHp-BTBHT0.2) in toluene (3 mL) and DMF (0.5 mL). For the synthesis of polymer acceptors N2200-BTBHTx, Pd2(dba)3 (3.0 mg) and tri(o-tolyl)phosphine (8.0 mg) were added to a degassed solution of NDIBr-C8C12(0.1–0.1x mmol), M1 (0.1× mmol), and 2ThSn (0.1 mmol) (x = 0 for N2200; x = 0.05 for N2200-BTBHT0.05; x = 0.1 for N2200-BTBHT0.1; x = 0.2 for N2200-BTBHT0.2) in toluene (5.5 mL). Then, the mixtures were stirred at 110 °C for 36 hours for PTzBI-EHp-BTBHTx and 18 h for N2200-BTBHTx, after which 2-(tributylstannyl)thiophene and 2-bromothiophene were sequentially added to the reaction with 2 h interval. After the mixture was cooled to room temperature, the product was precipitated in methanol and filtered. Then, the precipitate was purified by Soxhlet extraction with acetone, hexane, dichloromethane in sequence. The

dichloromethane fraction was collected and concentrated, which was then precipitated into methanol and then filtered. Finally, the solid precipitate was dried under vacuum for 48 h to remove the solvent. The polymer donors and acceptors were finally obtained as blue solid.

S1.3 J-V and EQE

The current density-voltage (J-V) characteristics of the devices were measured under 1 sun, AM 1.5 G solar simulator (Taiwan, Enlitech SS-F5) by using a computer-controlled Keithley 2400 Source Meter. The light intensity was calibrated by a China General Certification Center (CGC) certified reference silicon solar cell (Enlitech) before test, giving a 100 mW cm^{-2} light intensity during test. The external quantum efficiency (EQE) data were recorded with a QE-R test system (Enlitech).

S1.4 Light Operational Stability Measurements

Encapsulated Test: The devices were encapsulated with epoxy glue and glass in N_2 protected box. Then the devices were transferred into atmosphere, where the temperature was around 25 °C and humidity between 30%-40%, for the stability test. Light exposure was performed using a LED source with light intensity calibrated to achieve the same device performance measured by the standard AM 1.5 G solar simulator. Unencapsulated Test: Devices without encapsulation in temperature 25 °C or so, the 30%-40% of the atmosphere humidity stability test. Light exposure was performed using an LED light source and the light intensity was calibrated to achieve the same device performance as measured by a standard AM 1.5G solar simulator.

S1.5 Morphology Characterizations

AFM images were tested by a Digital Instrumental DI Multimode Nanoscope Ⅲ in a taping mode. TEM imagines were tested by a JEM-2100F instrument.

S2 Supplementary Figures and Tables

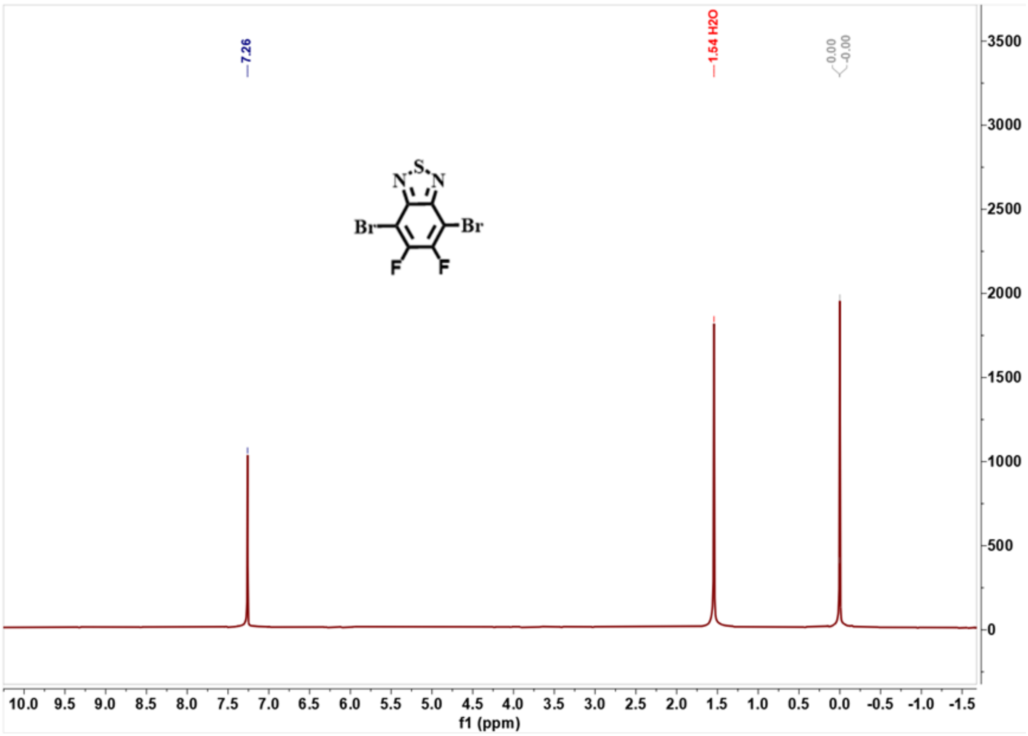

Fig. S1 ^{1}H NMR spectra of compound 2 ($CDCl_3$)

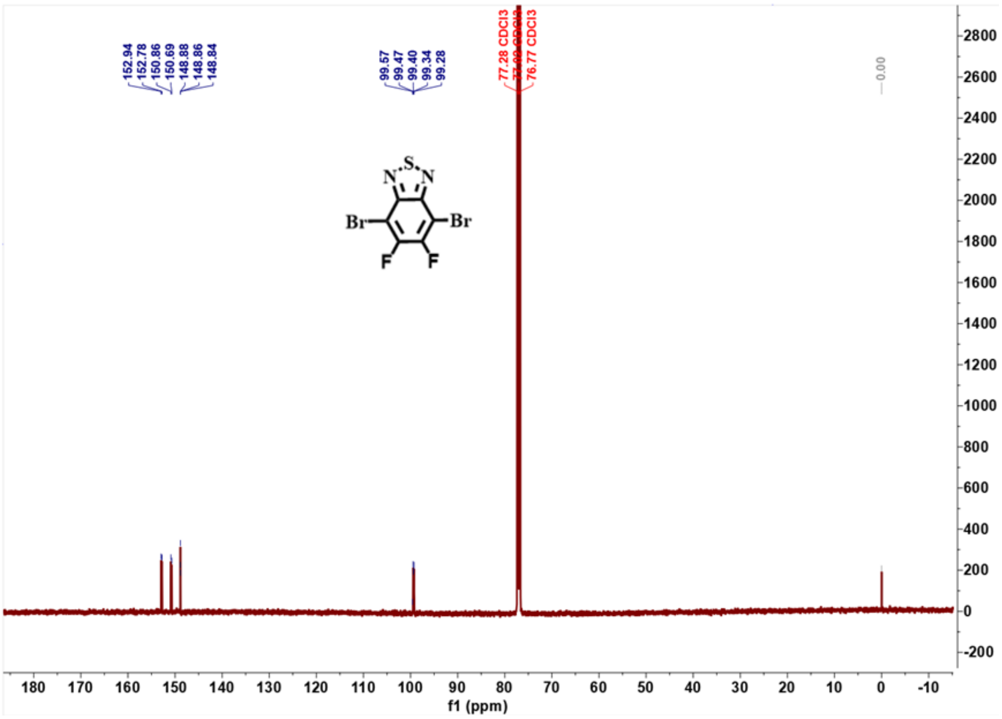

Fig. S2 ^{13}C NMR spectra of compound 2 ($CDCl_3$)

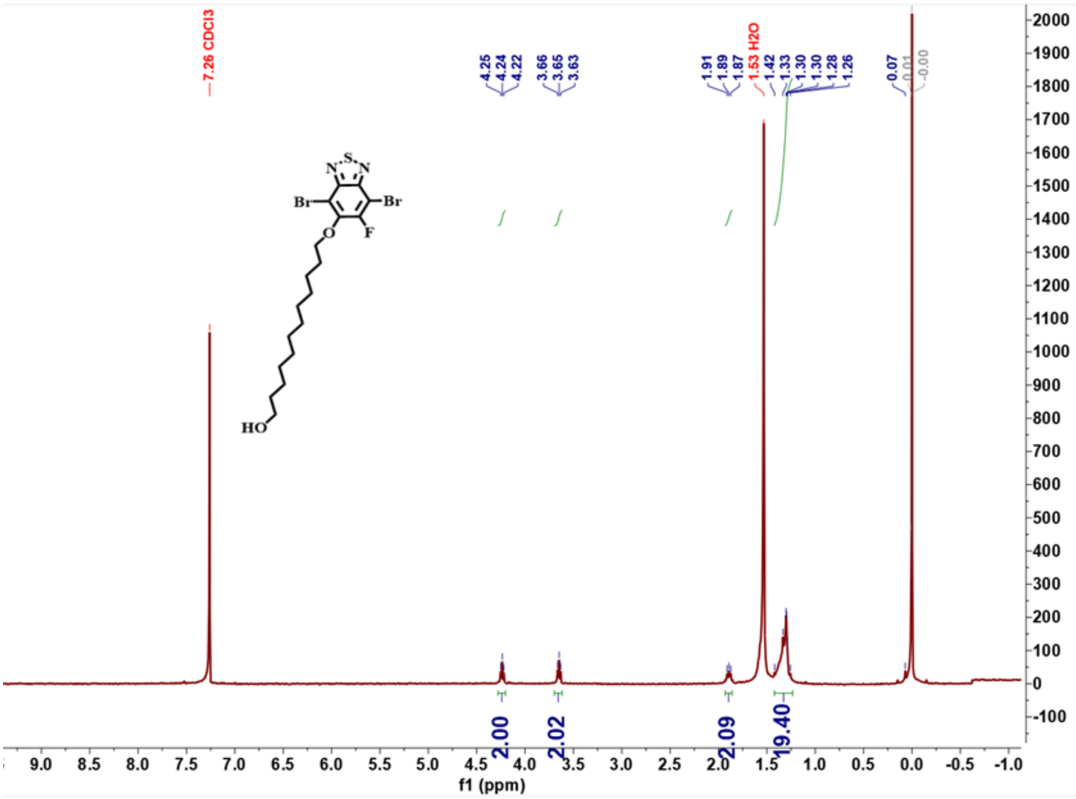

Fig. S3 ^{1}H NMR spectra of compound 3 ($CDCl_3$)

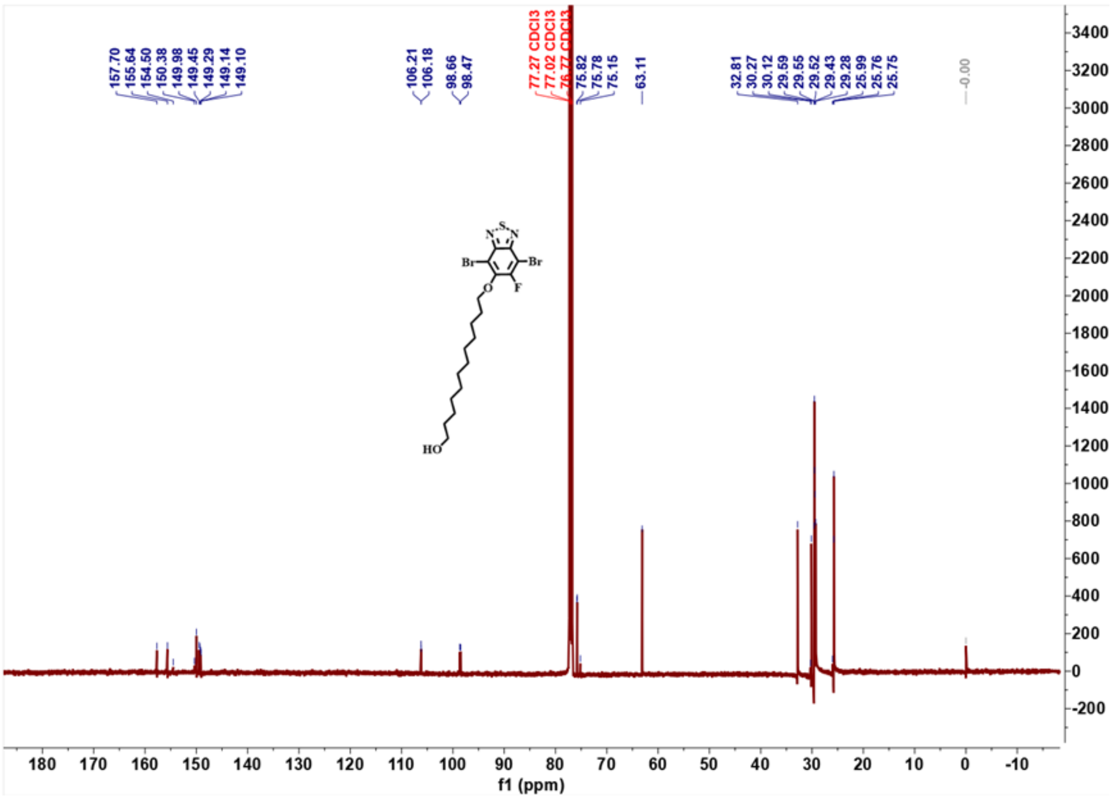

Fig. S4 ^{13}C NMR spectra of compound 3 ($CDCl_3$)

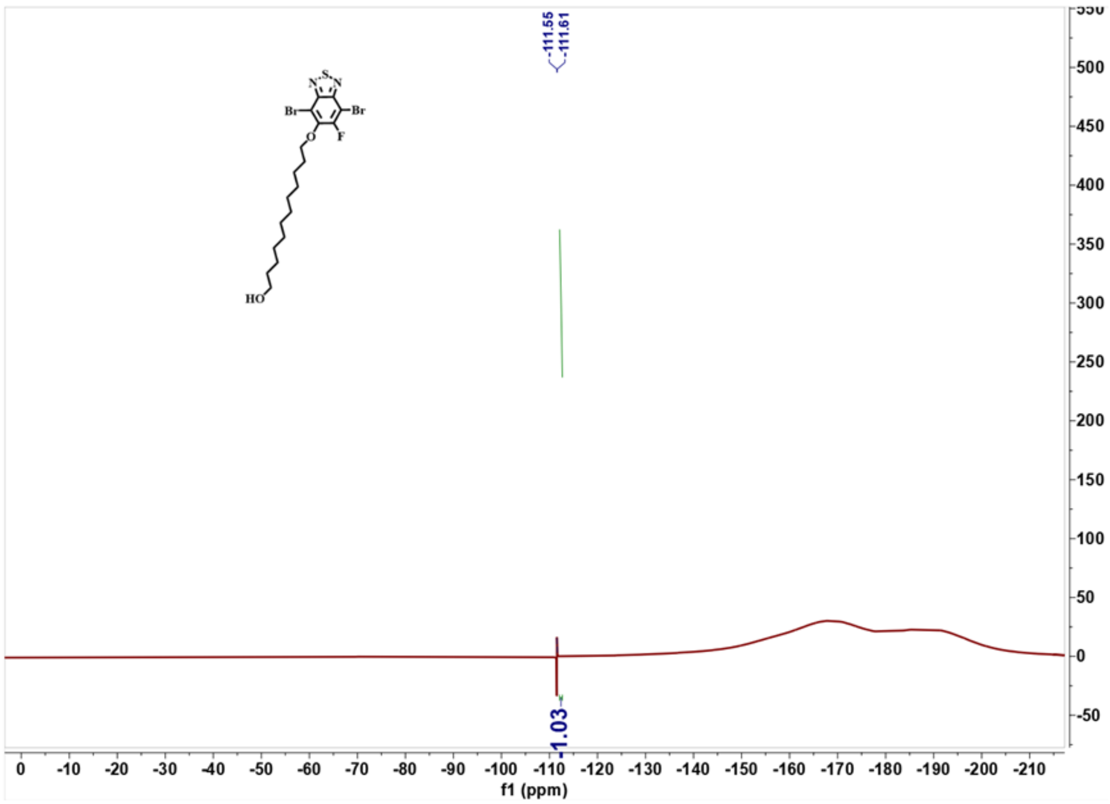

Fig. S5 ^{19}F NMR spectra of compound 3 ($CDCl_3$)

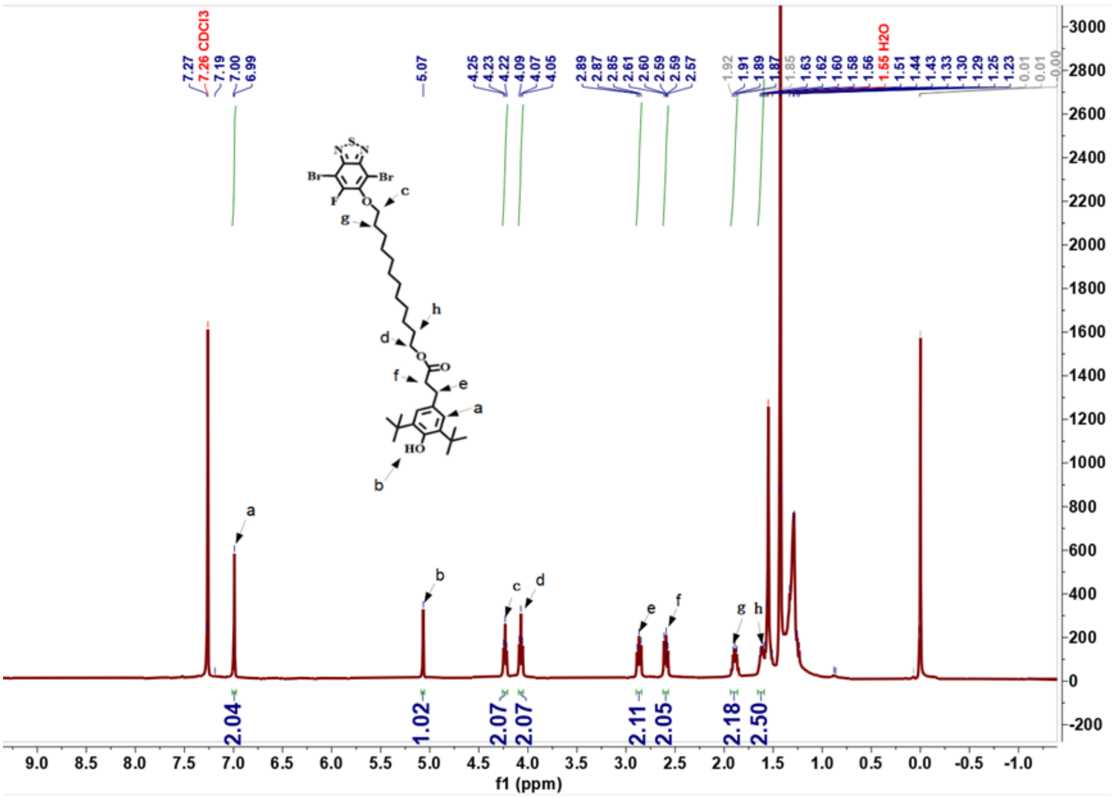

Fig. S6 ^{1}H NMR spectra of compound M1 ($CDCl_3$)

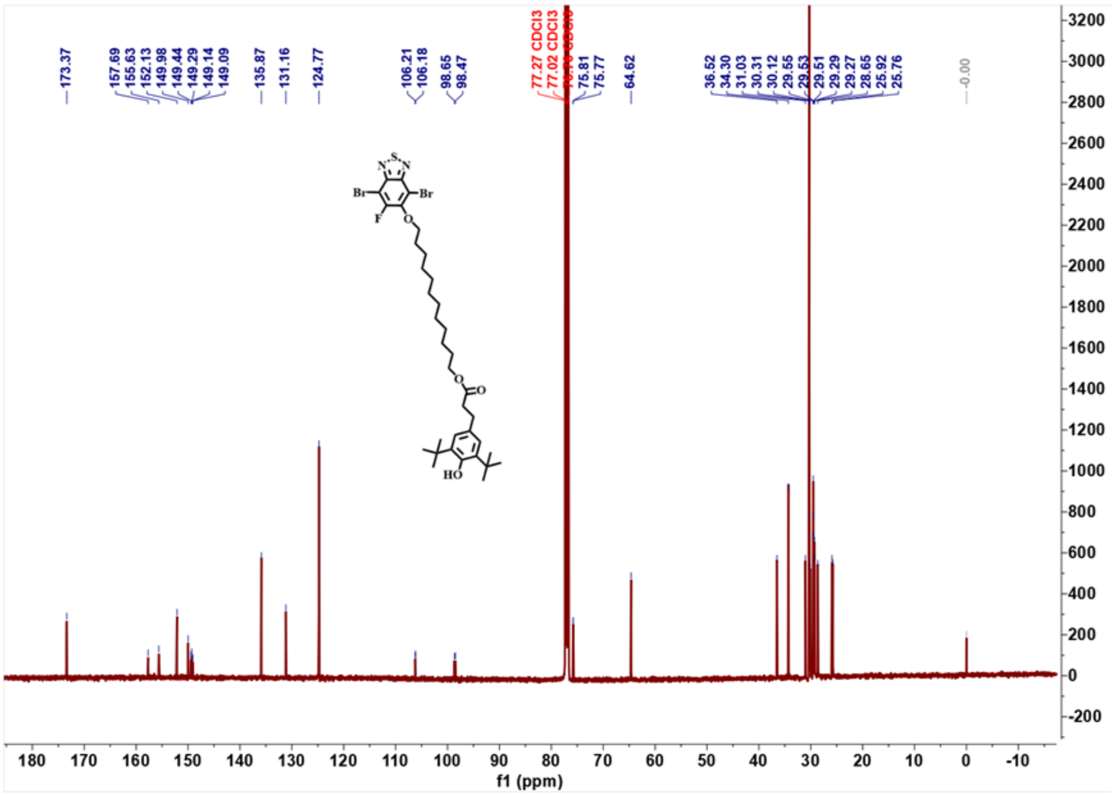

Fig. S7 ^{13}C NMR spectra of compound M1 ($CDCl_3$)

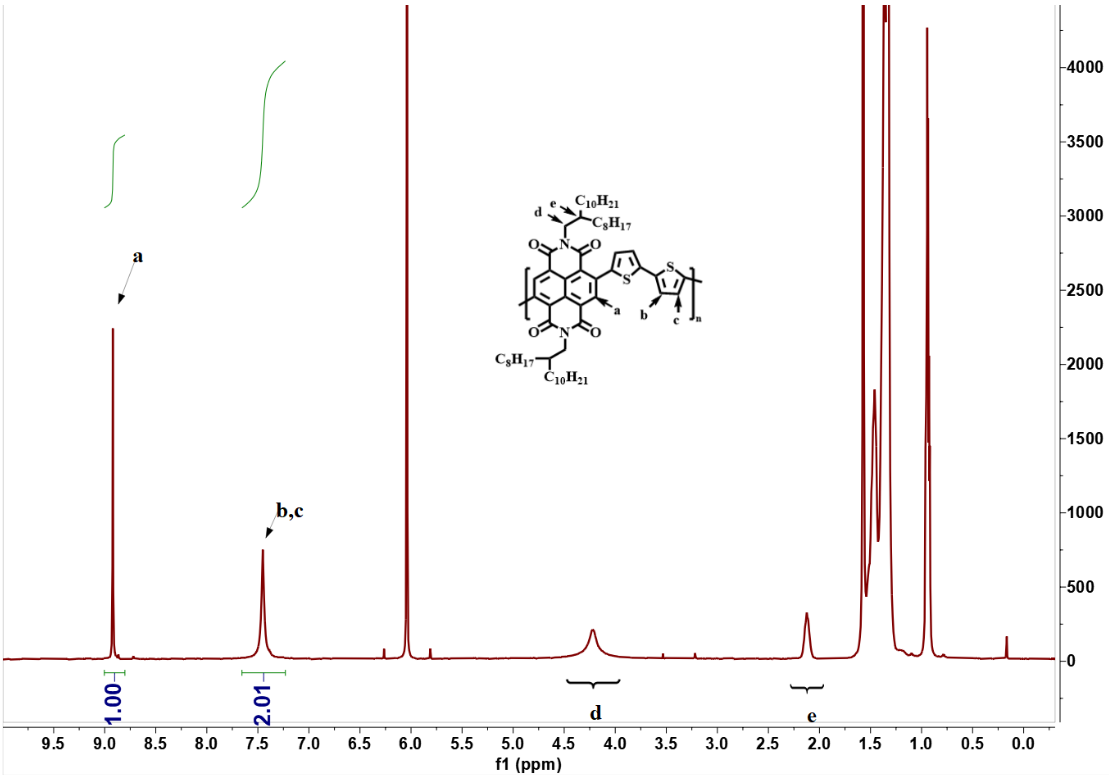

Fig. S8 ^{1}H NMR spectra of N2200 (1,1,2,2-$C_2D_2Cl_4$)

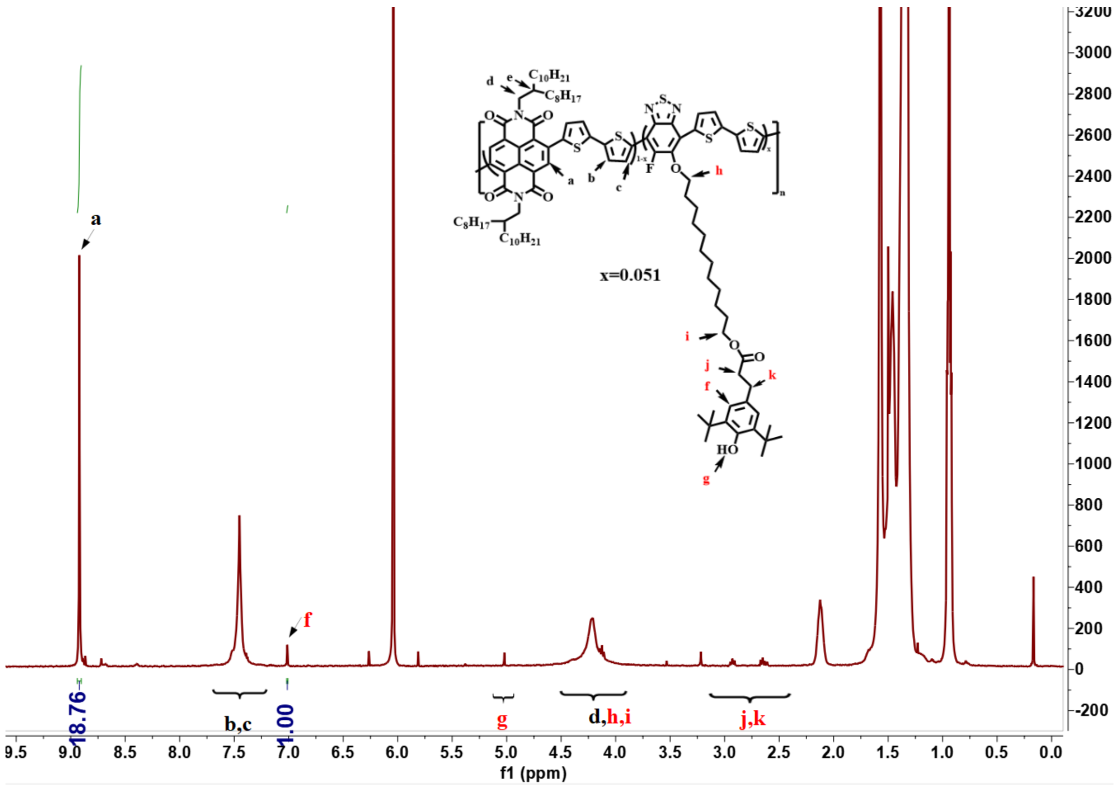

Fig. S9 ^{1}H NMR spectra of N2200-BTBHT0.05 (1,1,2,2-$C_2D_2Cl_4$)

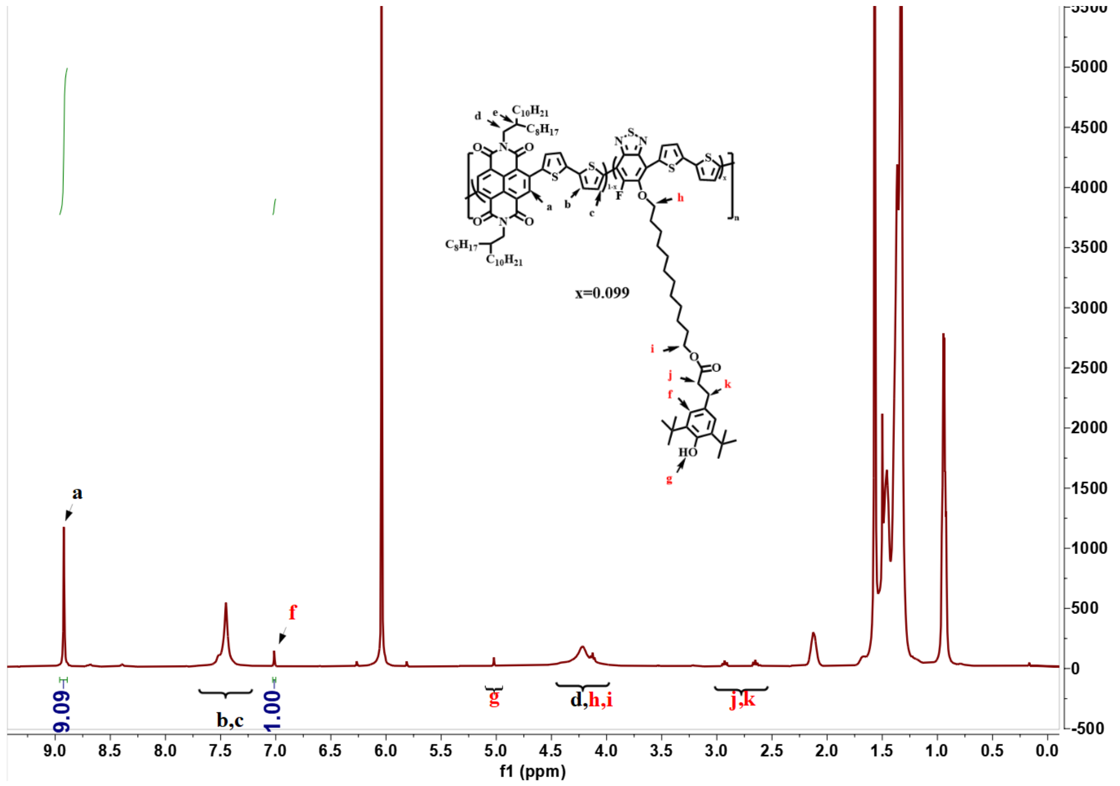

Fig. S10 ^{1}H NMR spectra of N2200-BTBHT0.1 (1,1,2,2-$C_2D_2Cl_4$)

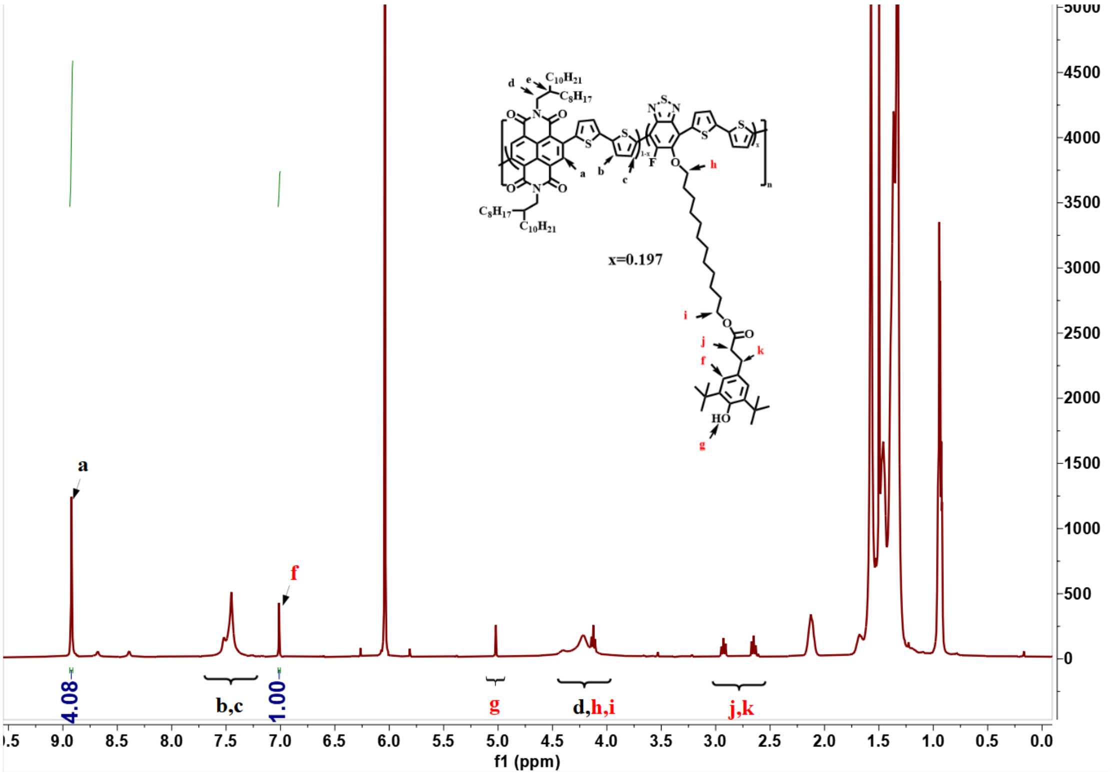

Fig. S11 ^{1}H NMR spectra of N2200-BTBHT0.2 (1,1,2,2-$C_2D_2Cl_4$)

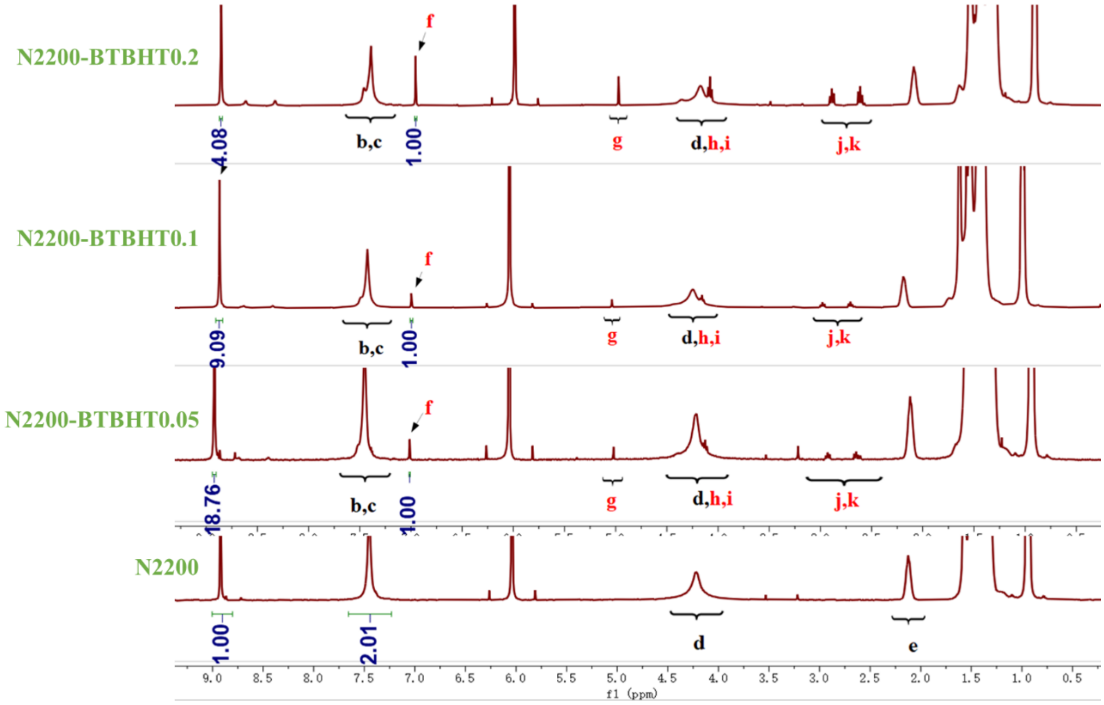

Fig. S12 Comparison of ^{1}H NMR spectra of N2200-BTBHTx

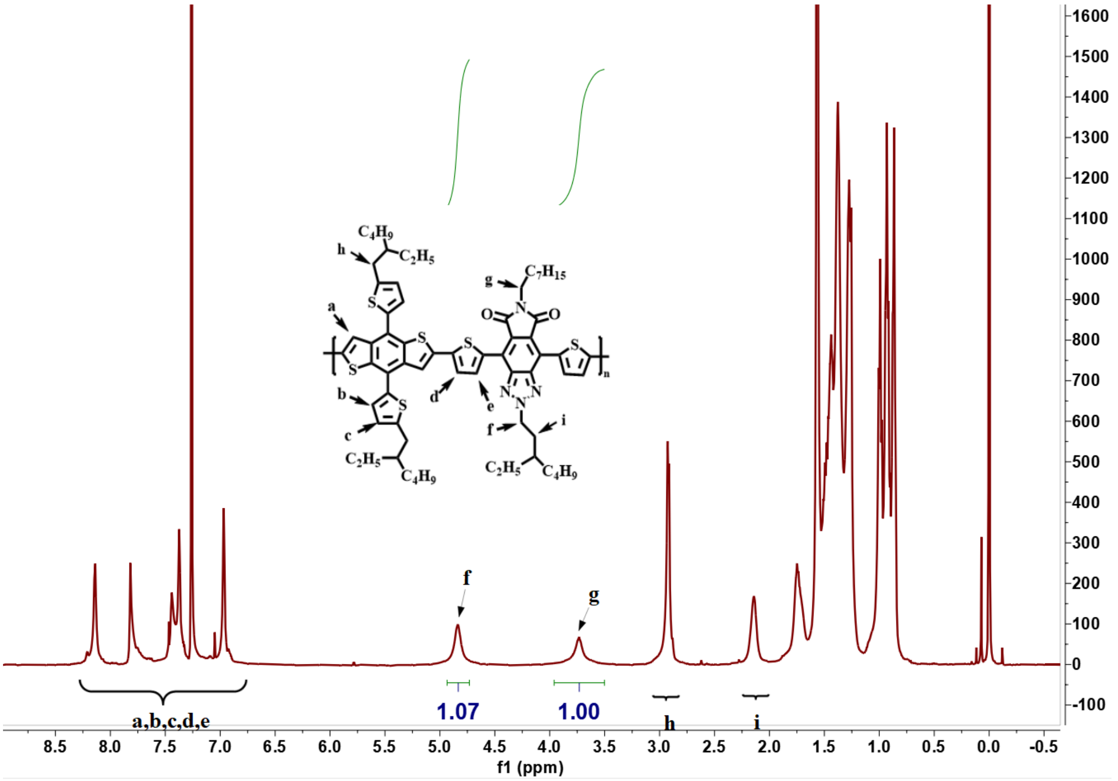

Fig. S13 ^{1}H NMR spectra of PTzBI-EHp ($CDCl_3$)

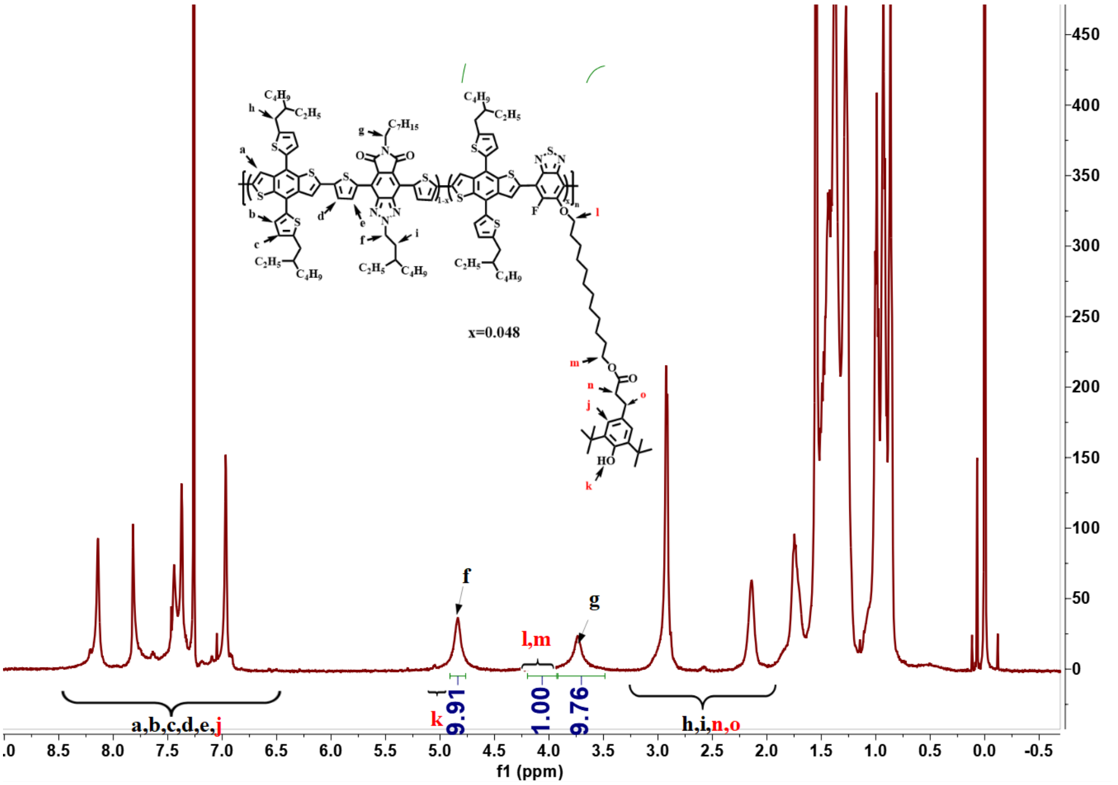

Fig. S14 ^{1}H NMR spectra of PTzBI-EHp-BTBHT0.05 ($CDCl_3$)

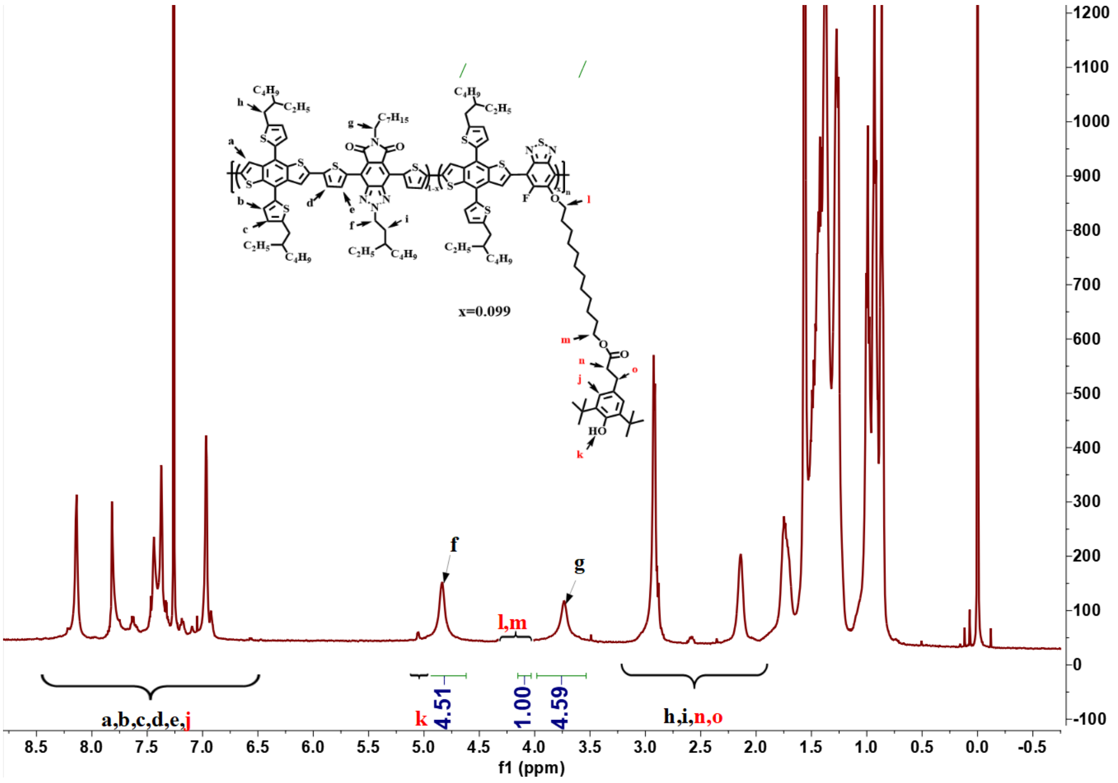

Fig. S15 ^{1}H NMR spectra of PTzBI-EHp-BTBHT0.1 ($CDCl_3$)

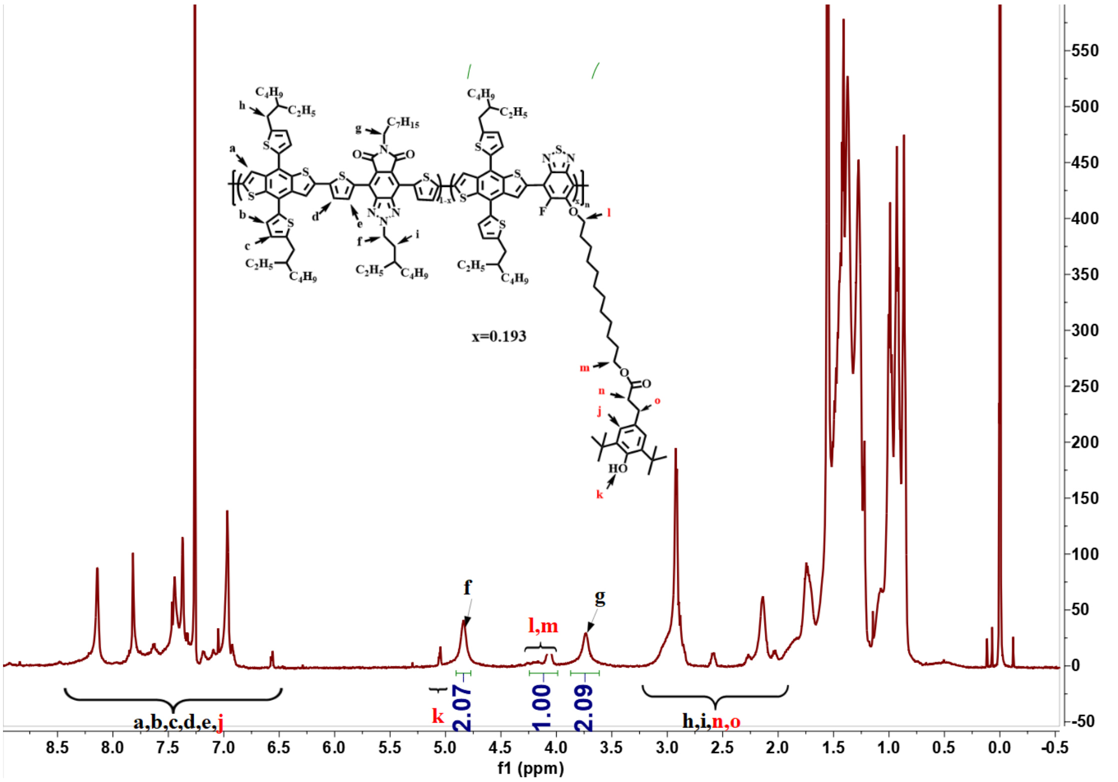

Fig. S16 ^{1}H NMR spectra of PTzBI-EHp-BTBHT0.2 ($CDCl_3$)

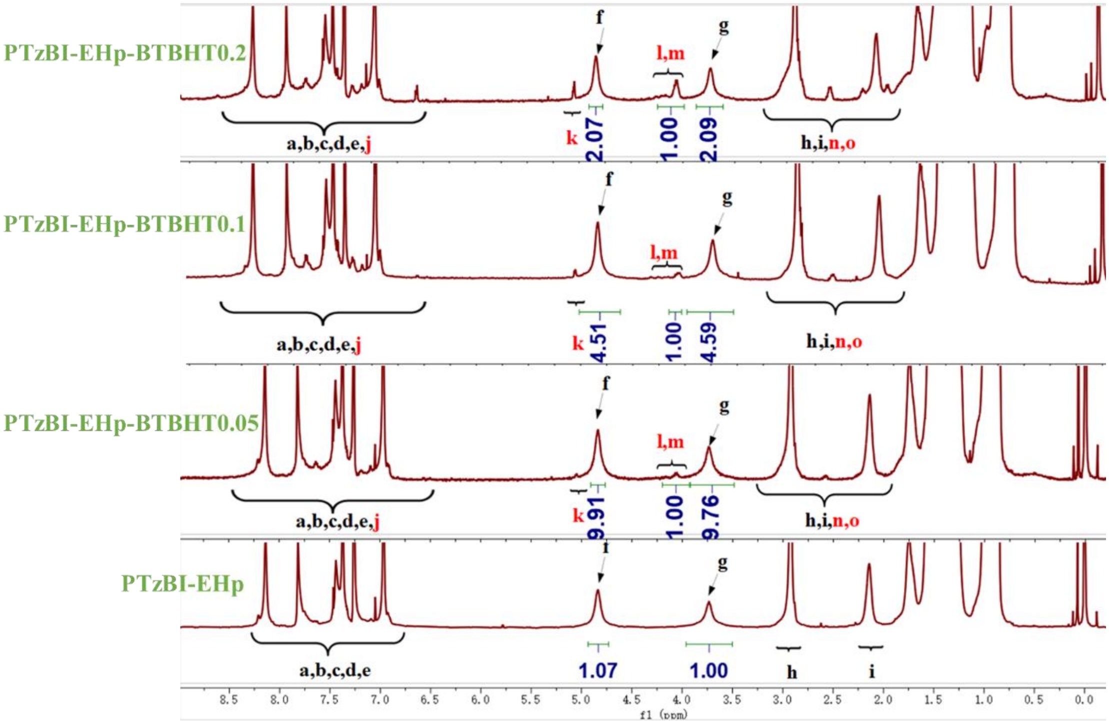

Fig. S17 Comparison of ^{1}H NMR spectra of PTzBI-EHp-BTBHTx

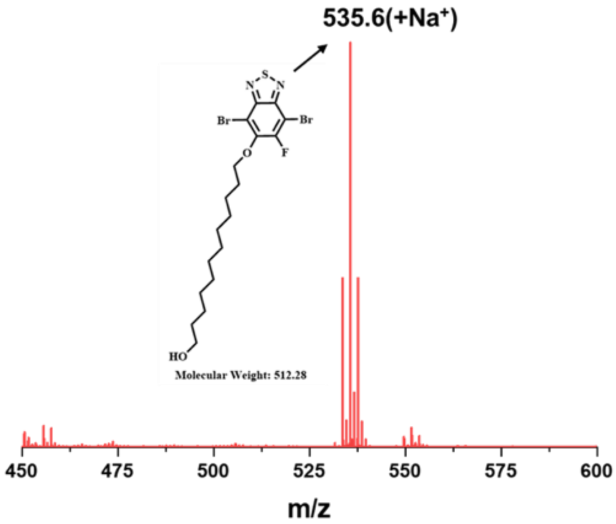

Fig. S18 The mass spectral data of compound 3

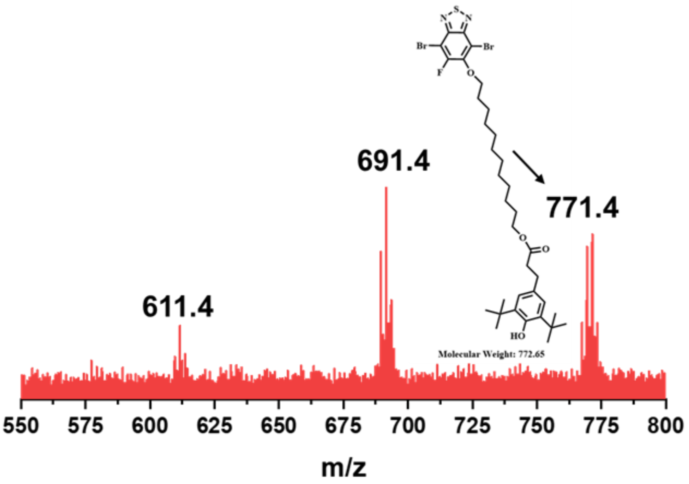

Fig. S19 The mass spectral data of compound M1

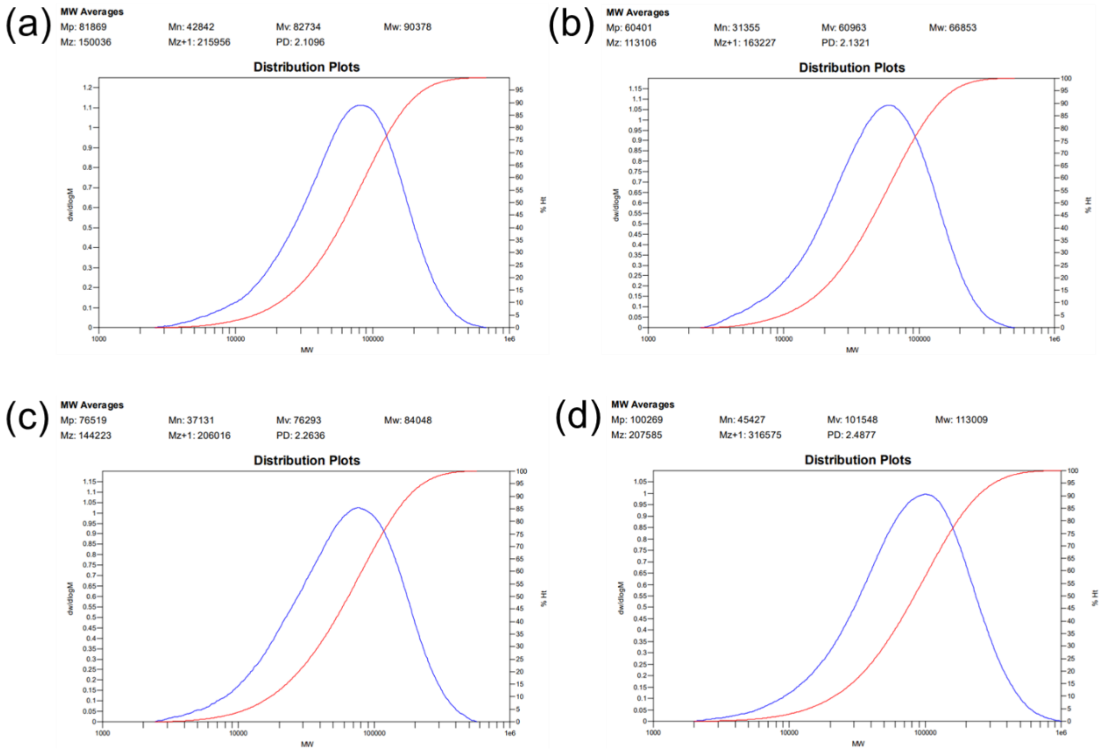

Fig. S20 The gel permeation chromatography (GPC) of **(a)** PTzBI-EHp, **(b)** PTzBI-EHp-BTBHT0.05, **(c)** PTzBI-EHp-BTBHT0.1 and **(d)** PTzBI-EHp-BTBHT0.2

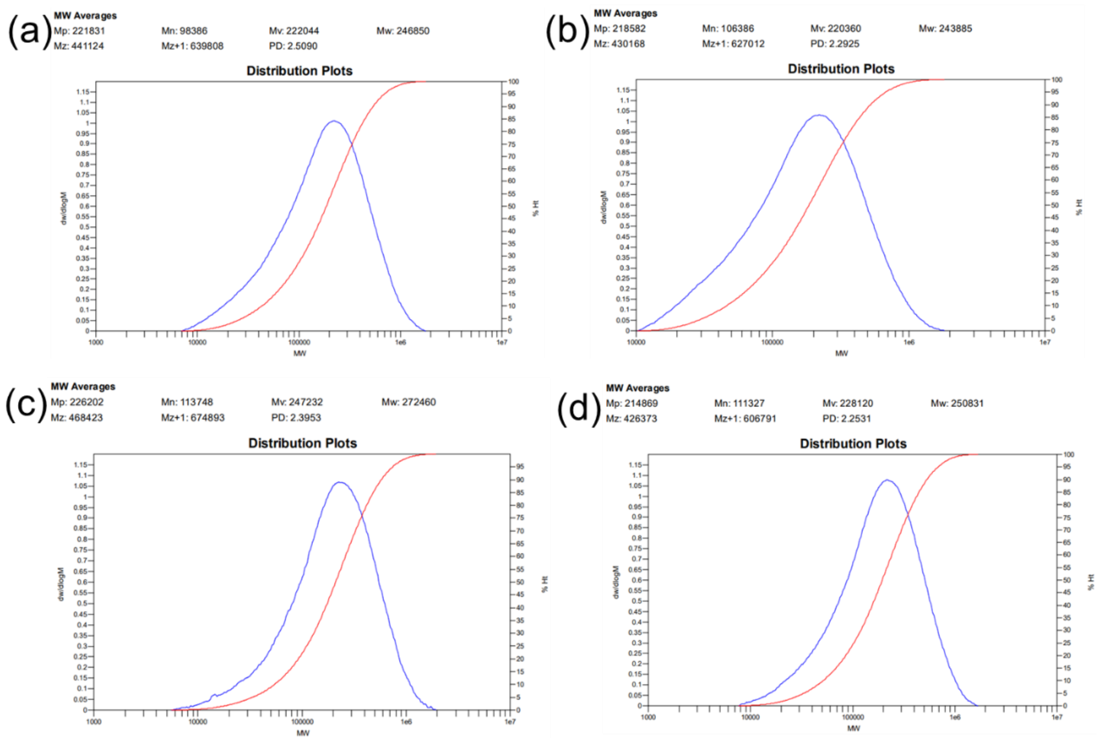

Fig. S21 The gel permeation chromatography (GPC) of **(a)** N2200, **(b)** N2200-BTBHT0.05, **(c)** N2200-BTBHT0.1 and **(d)** N2200-BTBHT0.2

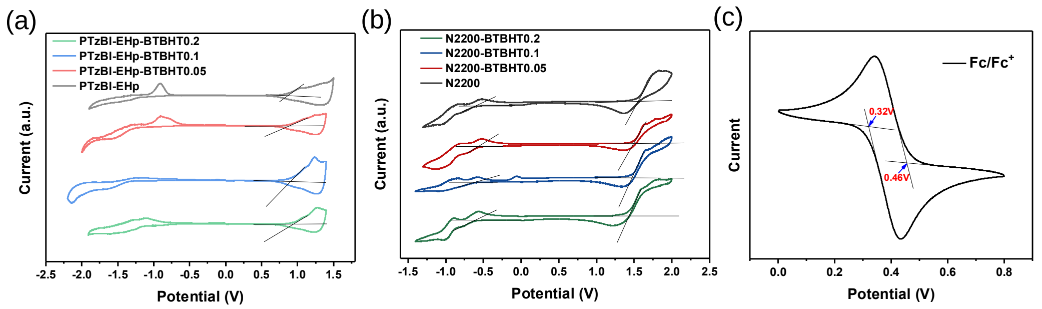

Fig. S22 Cyclic voltammetry curves of **(a)** polymer donors PTzBI-EHp-BTBHTx (x = 0, 0.05, 0.1, 0.2), **(b)** polymer acceptors N2200-BTBHTx (x = 0, 0.05, 0.1, 0.2) and **(c)** ferrocene/ferrocenium

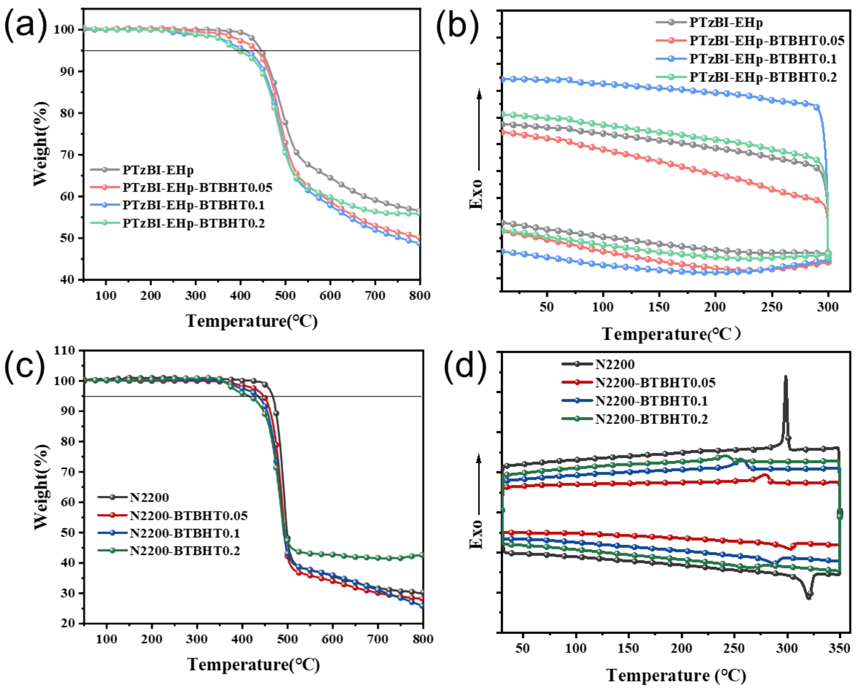

Fig. S23 (a, c) Thermal gravimetric analysis of polymer donors PTzBI-EHp-BTBHTx (x = 0, 0.05, 0.1, 0.2) and polymer acceptors N2200-BTBHTx (x = 0, 0.05, 0.1, 0.2), respectively. **(b, d)** DSC traces of polymer donors and polymer acceptors, respectively

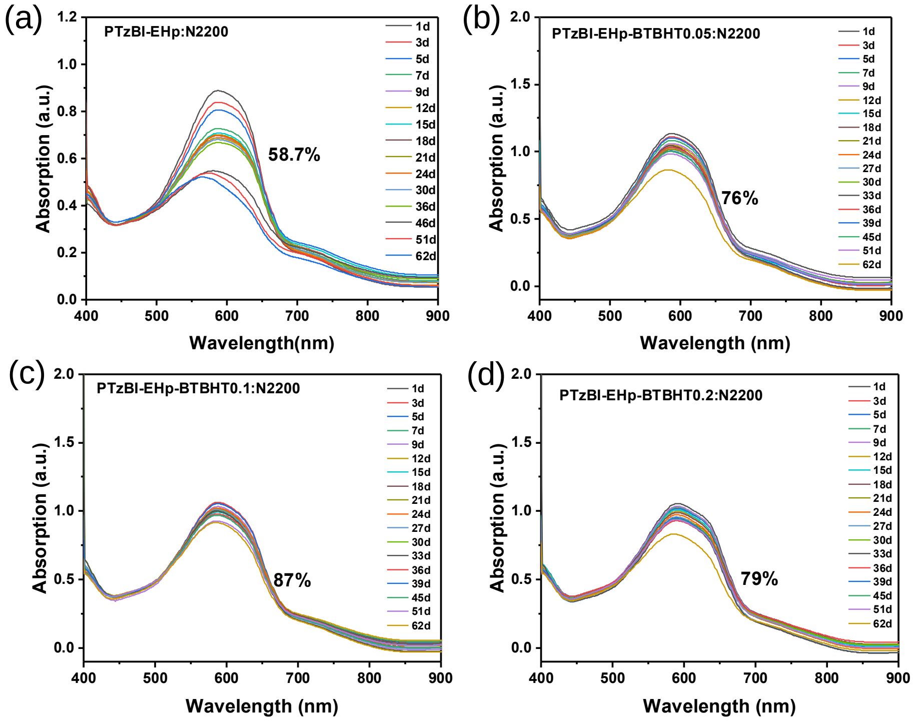

Fig. S24 UV-vis-NIR absorption spectra of PTzBI-EHp-BTBHTx: N2200 films under light and ambient condition; **(a)** PTzBI-EHp, **(b)** PTzBI-EHp-BTBHT0.05, **(c)** PTzBI-EHp-BTBHT0.1, and **(d)** PTzBI-EHp-BTBHT0.2

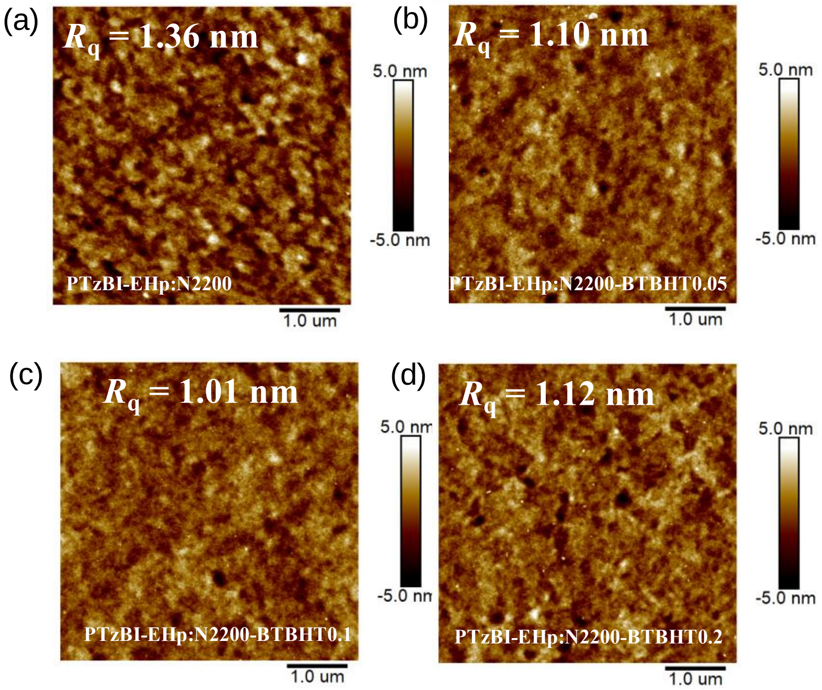

Fig. S25 (a-d) AFM images of blend films of PTzBI-EHp: N2200-BTBHTx (x = 0, 0.05, 0.1, 0.2)

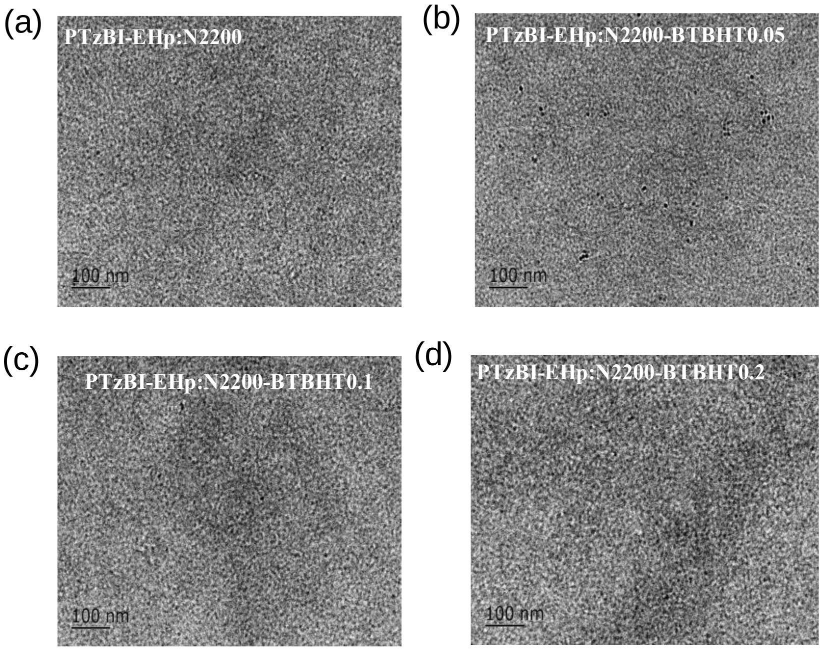

Fig. S26 (a-d) The TEM images of films of PTzBI-EHp: N2200-BTBHTx (x = 0, 0.05, 0.1, 0.2)

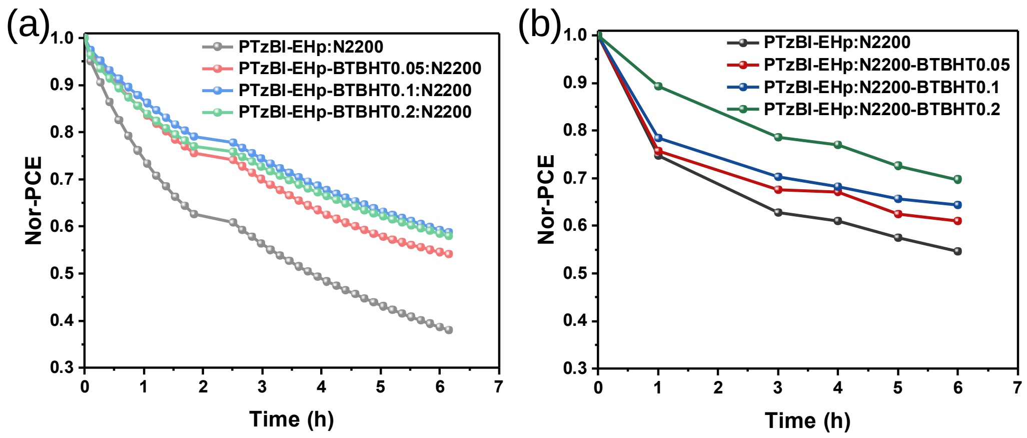

Fig. S27 (a) Evolution of PCE of PTzBI-EHp-BTBHTx: N2200 (x = 0, 0.05, 0.1, 0.2) cells aged at mpp with continuous light illumination and ambient condition; **(b)** Evolution of PCE of PTzBI-EHp: N2200-BTBHTx (x = 0, 0.05, 0.1, 0.2) cells aged with continuous light illumination and ambient condition

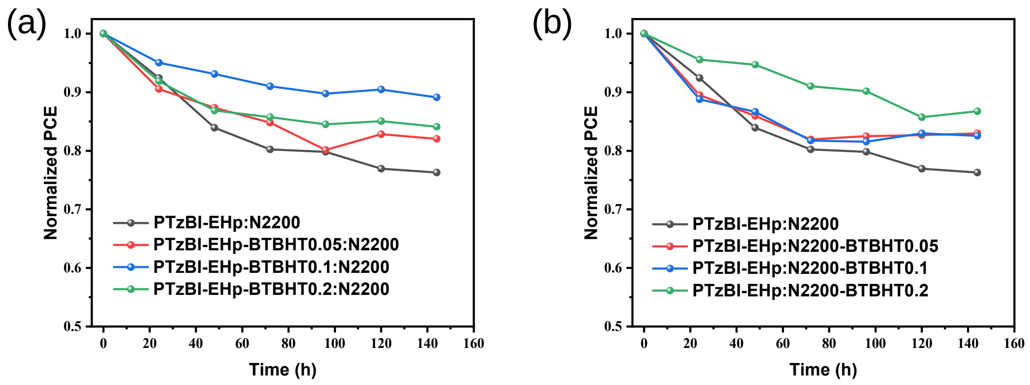

Fig. S28 The PCE track under 80℃ at inert atmosphere for encapsulated all-PSCs based on **(a)** PTzBI-EHp-BTBHTx (x = 0, 0.05, 0.1, 0.2): N2200 and **(b)** PTzBI-EHp: N2200-BTBHTx (x = 0, 0.05, 0.1, 0.2), respectively

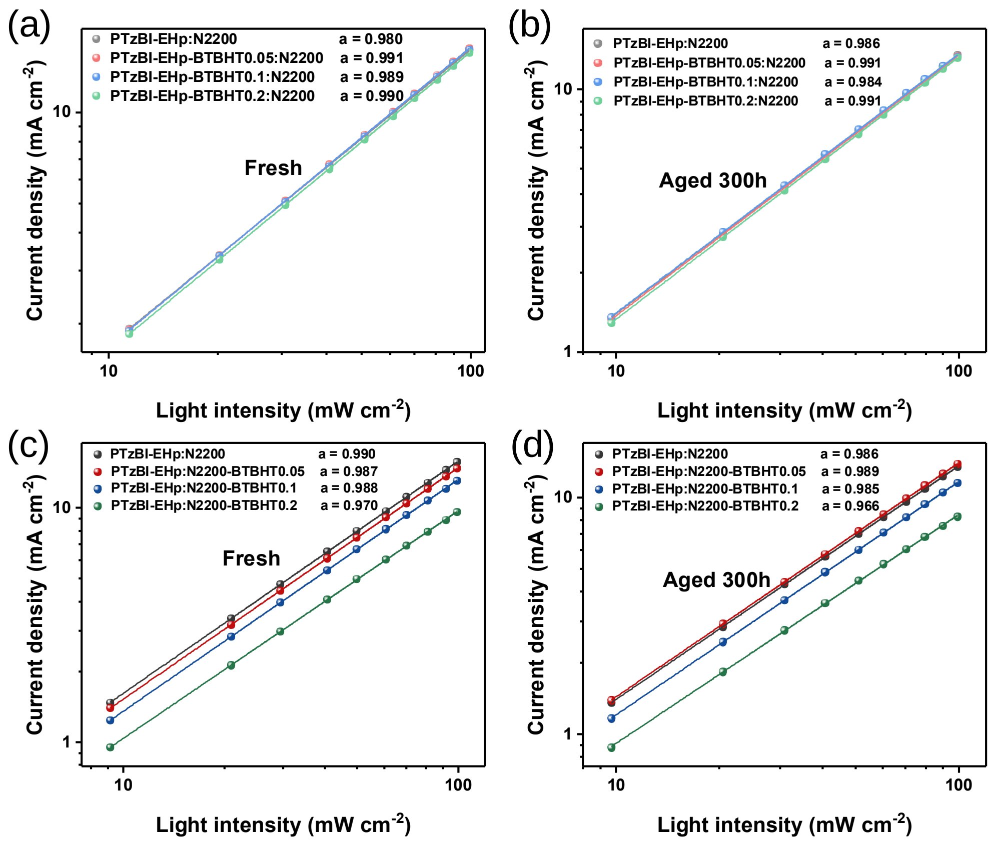

Fig. S29 (a) Fresh and **(b)** aged 300 h light intensity dependence of J_{sc} of the PTzBI-EHp-BTBHTx: N2200 (x = 0, 0.05, 0.1, 0.2) cells; **(c)** Fresh and **(d)** aged 300 h light intensity dependence of J_{sc} of the PTzBI-EHp: N2200-BTBHTx (x = 0, 0.05, 0.1, 0.2) cells

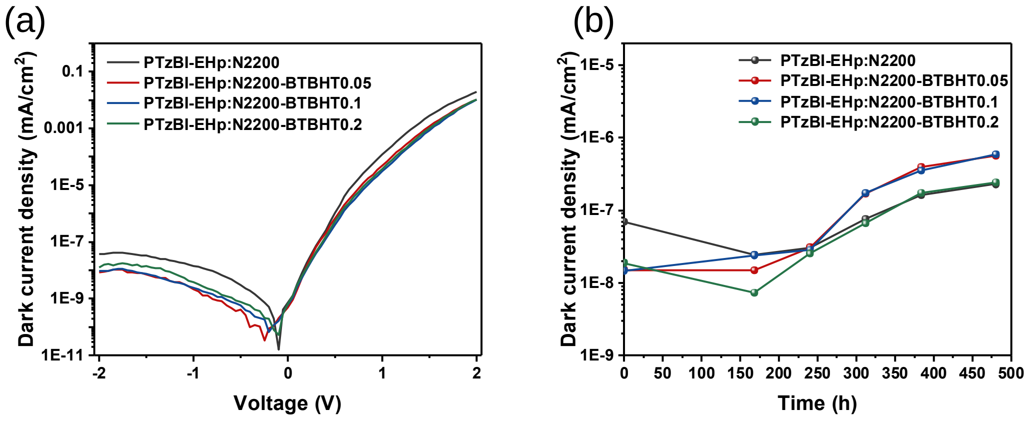

Fig. S30 (a) *J-V* curves of the OPDs based on polymer acceptors N2200-BTBHTx (x = 0, 0.05, 0.1, 0.2) under dark condition; **(b)** The stability of J_d of corresponding OPDs after aging for several days

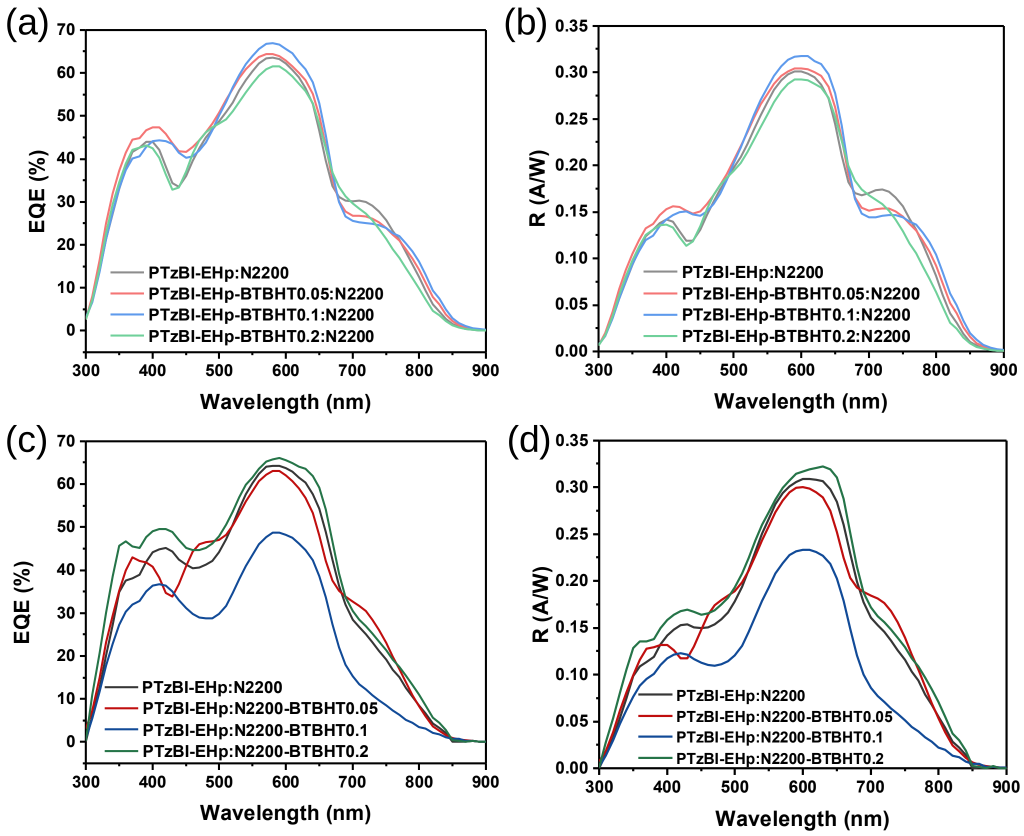

Fig. S31 (a, c) EQE curves and **(b, d)** spectral responsivity at zero bias of OPDs

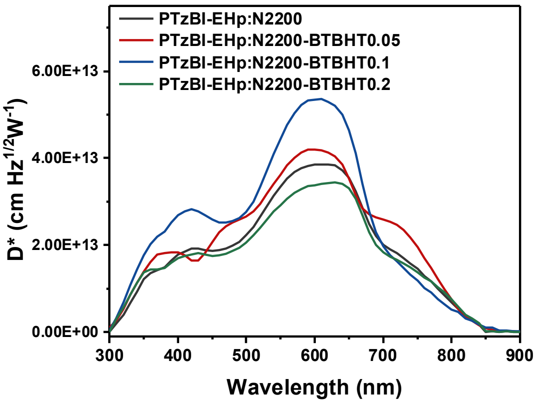

Fig. S32 Specific detectivity obtain from J_d of OPDs based on PTzBI-EHp: N2200-BTBHTx (x = 0, 0.05, 0.1, 0.2)

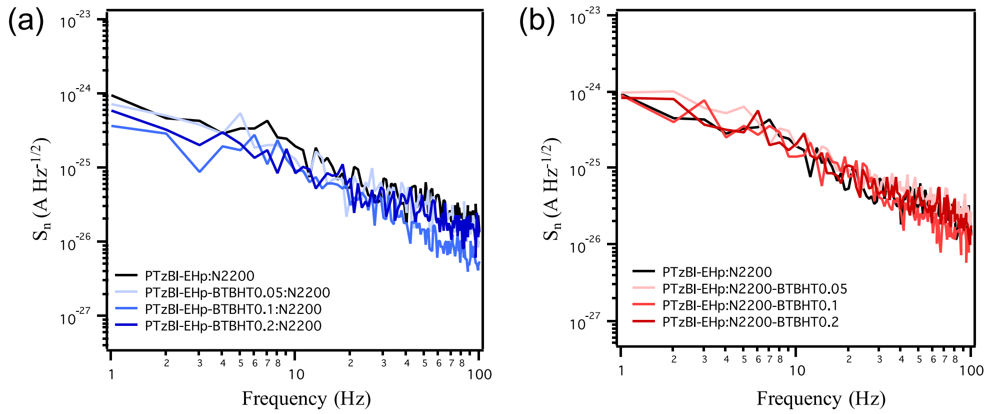

Fig. S33 Actual noise (at −0.1V) curves of OPDs based on **(a)** PTzBI-EHp-BTBHTx: N2200 (x = 0, 0.05, 0.1, 0.2) and **(b)** PTzBI-EHp: N2200-BTBHTx (x = 0, 0.05, 0.1, 0.2), respectively, after aging

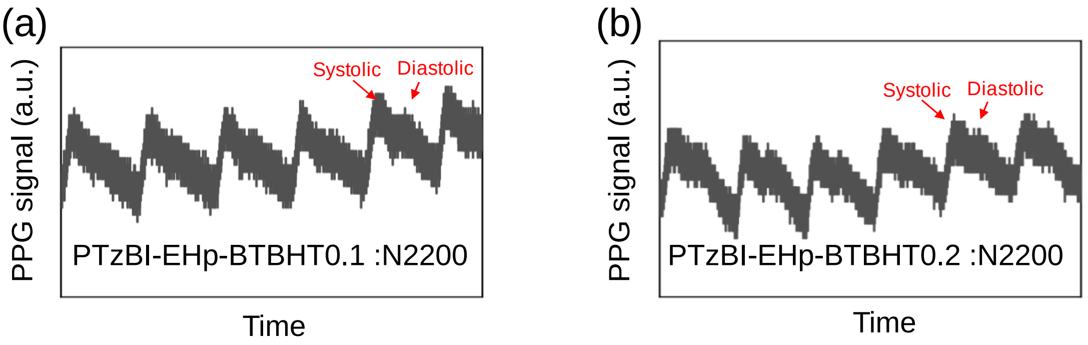

Fig. S34 (a, b) PPG signal fluctuations of OPD devices after aging

Table S1 Photovoltaic parameters of all-PSCs

BHJ	V_{oc} (V)	J_{sc} (mA cm^{-2})	FF (%)	PCE$_{MAX}$ (%)
PTzBI-EHp-BTBHT0.1:	0.85±0.00	14.87±0.16	69.10±0.74	8.72±0.10
N2200- BTBHT0.05	(0.85)	(14.97)	(69.83)	(8.88)
PTzBI-EHp-BTBHT0.2:	0.86±0.00	14.91±0.13	65.97±0.86	8.44±0.19
N2200- BTBHT0.05	(0.86)	(15.10)	(66.32)	(8.61)
PTzBI-EHp-BTBHT0.1:	0.85±0.00	13.98±0.58	65.55±1.08	7.81±0.19
N2200- BTBHT0.1	(0.85)	(14.53)	(64.83)	(8.04)
PTzBI-EHp-BTBHT0.2:	0.86±0.00	13.54±0.23	62.22±1.85	7.24±0.17
N2200- BTBHT0.1	(0.86)	(13.79)	(13.77)	(7.45)

Table S2 The exciton dissociation probability P(E, T) values and the exciton dissociation probability (G_{max}) of OSCs

BHJ	PTzBI-EHp:N2200	PTzBI-EHp-BTBHT0.05:N2200	PTzBI-EHp-BTBHT0.1:N2200	PTzBI-EHp-BTBHT0.2:N2200	PTzBI-EHp:N2200-BTBHT0.05	PTzBI-EHp:N2200-BTBHT0.1	PTzBI-EHp:N2200-BTBHT0.2
P(E, T) (%)	93.58	94.14	96.28	95.95	92.04	89.33	81.87
G_{max} (m^{-3} s^{-1})	1.06×10^{28}	1.03×10^{28}	1.06×10^{28}	1.04×10^{28}	1.04×10^{28}	8.69×10^{27}	6.89×10^{27}

Table S3 Shut resistance values of OPDs

BHJ	PTzBI-EHp:N2200	PTzBI-EHp-BTBHT0.05:N2200	PTzBI-EHp-BTBHT0.1:N2200	PTzBI-EHp-BTBHT0.2:N2200	PTzBI-EHp:N2200-BTBHT0.05	PTzBI-EHp:N2200-BTBHT0.1	PTzBI-EHp:N2200-BTBHT0.2
R_{sh} (Ω cm^2)	2.0×10^8	4.6×10^8	5.0×10^8	4.0×10^8	5.3×10^8	3.1×10^8	2.5×10^8

Note S1

The total noise of a photodiode contains shot noise (S_{shot}), thermal noise (S_{thermal}), 1/f noise ($S_{1/\mathrm{f}}$), and generation-recombination noise ($S_{\mathrm{g-r}}$) components. The noise current can be expressed as follows:

$$S_{\mathrm{noise}} = \sqrt{S^2{}_{\mathrm{shot}} + S^2{}_{\mathrm{thermal}} + S^2{}_{1/\mathrm{f}} + S^2{}_{\mathrm{g-r}}}$$

The theoretical S_{shot} can be described by Equation

$$S_{\mathrm{sh}} = \frac{i_{\mathrm{sh}}}{\sqrt{B}} = \sqrt{2qJ_{\mathrm{d}}}$$

When the shot noise is dominant, the specific detectivity (D*) can be described by Equation

$$D^* = \frac{R_{\mathrm{res}}\sqrt{A}}{S_{\mathrm{noise}}} = \frac{R_{\mathrm{res}}}{\sqrt{2qJ_{\mathrm{d}}}}$$
